# Hidden social and emotional competencies in autism spectrum disorders captured through the digital lens

**DOI:** 10.3389/fpsyt.2025.1559202

**Published:** 2025-04-07

**Authors:** Elizabeth B. Torres, Joe Vero, Neel Drain, Richa Rai, Theodoros Bermperidis

**Affiliations:** ^1^ Psychology Department, Sensory Motor Integration Laboratory, Rutgers University, Piscataway, NJ, United States; ^2^ Computer Science Department, Center for Biomedicine Imaging and Modeling (CBIM), Rutgers University, Piscataway, NJ, United States; ^3^ Rutgers University Center for Cognitive Science (RUCCS), Piscataway, NJ, United States

**Keywords:** facial micro-expressions, autism spectrum disorders, emotions, stochastic analysis, motor control, automatic screening

## Abstract

**Background/objectives:**

The current deficit model of autism leaves us ill-equipped to connect with persons on the spectrum, thus creating disparities and inequalities in all aspects of social exchange in which autistic individuals try to participate. Traditional research models also tend to follow the clinical definition of impairments in social communication and emotions without offering personalized therapeutic help to autistic individuals. There is a critical need to redefine autism with the aim of co-adapting and connecting with this exponentially growing sector of society. Here, we hypothesize that there are social and emotional competencies hidden in the movements’ nuances that escape the naked eye. Further, we posit that we can extract such information using highly scalable means such as videos from smartphones.

**Methods:**

Using a phone/tablet app, we recorded brief face videos from 126 individuals (56 on the spectrum of autism) to assess their facial micro-motions during several emotional probes in relation to their resting state. We extracted the micro-movement spikes (MMSs) from the motion speed along 68 points of the OpenFace grid and empirically determined the continuous family of probability distribution functions best characterizing the MMSs in a maximum likelihood sense. Further, we analyzed the action units across the face to determine their presence and intensity across the cohort.

**Results:**

We find that the continuous Gamma family of probability distribution functions describes best the empirical face speed variability and offers several parameter spaces to automatically classify participants. Unambiguous separation at rest denotes marked differences in stochastic patterns between neurotypicals and autistic individuals amenable to further separate autistic individuals according to the required level of support. Both groups have comparable action units present during emotional probes. They, however, operate within parameter ranges that fall outside our perceptual umwelt and, as such, do not meet our expectations from prior experiences. We cannot detect them.

**Conclusions:**

This work offers new methods to detect hidden facial features and begin the path of augmenting our perception to include those signatures of the autism spectrum that can enhance our capacity for social interactions, communication, and emotional support to meet theirs.

## 1 Introduction

The current societal perception of individuals with autism spectrum disorders (ASD) inherently depends on the diagnostic criteria and the research narrative that such criteria advance (1). Every paper on autism starts with the description of ASD as deficits in social interactions and communication (2, 3). There are no metrics indicative of readiness potential to socially interact or to communicate, despite the recognized plasticity of the developing nervous system (4).

The screening and diagnostic process of neurodevelopmental disorders relies exclusively on observation, so it remains a challenge to identify any potential competencies for social and emotional exchange. Surely, the system survived a developmental insult and learned to function in the world over the period of 3–4 years that, on average, takes to receive such a diagnosis (5). During this time, it is possible that coping mechanisms developed without the type of support that would be required to gradually learn to bridge the mental intent of a person to the physical execution of a person’s goal-directed thoughts into congruent actions under volitional control (6, 7). The disconnect that autistic individuals report between mental intent and physical action may mislead us, as we have already built-in certain expectations and biases from interactions with others whose actions match their intended consequences.

Because large portions of these aspects of motor control constitute behavioral nuances that transpire largely beneath awareness, we tend to miss them when relying on visual observation alone. Blurred among our expectations and biased subjective experiences may be those competencies of the autistic system longing to build social rapport (8). We may be missing the opportunity to truly help and connect with autistic individuals by relying exclusively on observation.

Observational instruments in the USA, like the Autism Diagnostic Observation Schedule (ADOS; currently in version 2; 2) and the Diagnostic and Statistical Manual of Mental Disorders (DSM; currently in version 5, from the American Psychiatric Association (9)) are built upon subjective opinion. Both instruments are consistently and broadly used in basic research to drive research questions that would eventually be translated into practice. They are also used in clinical practices to recommend services and to drive funding and societal support for this exponentially growing sector of the population.

**Figure 1:**
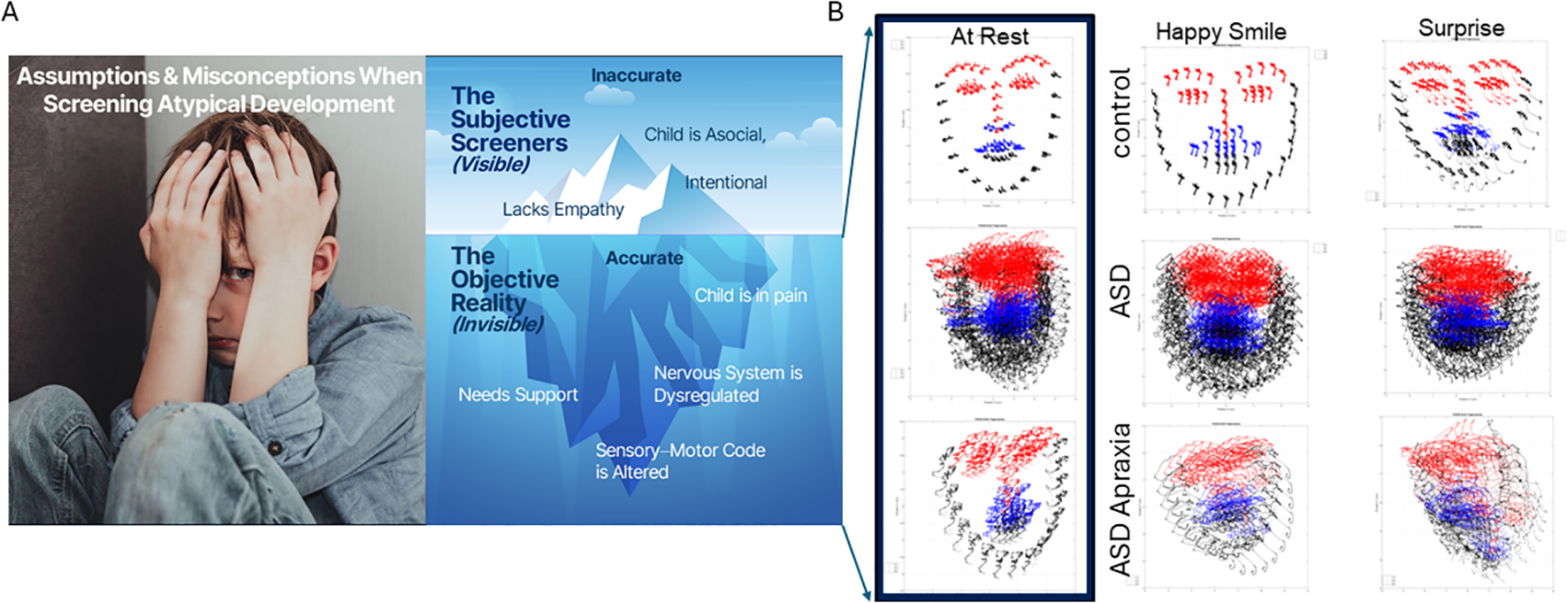
Current deficit model of autism **(A)** is the tip of the iceberg. New advances in computer vision amid a digital revolution begin to reveal a new picture of autism **(B)** whereby the child needs personalized help and objective science-based support to thrive. Here, 5 seconds of video data captured at rest; smiling and surprised show the dysregulated facial micro motions of the child, surely impacting visual perception of others in the social scene and having heightened uncertainty from a brain that is receiving excessive random noise as reafferent input. Under such uncertain world, anxiety and dysregulated states of pain and hypervigilance are not uncommon in ASD. ASD, autism spectrum disorder.

These tools, which rely exclusively on observation (10), have led to several prominent theories of autism (11, 12), which in turn, have resulted in stigma and even denial of basic education rights to some autistic individuals in the USA. The very notion that autistic individuals lack empathy (13, 14), lack the social desire to communicate (15, 16), are deviant or do not have a theory of mind (12, 17), and, more generally, are deprived of human emotions (18–20) has systematically contributed to a narrative that negatively impacts these people’s lives (1, 8). The science behind such opinions is grounded in subjective observational techniques. It merely exposes the tip of the iceberg in a rather misleading way (**Figure 1A**). The physiological underpinnings of what we observe and interpret are starting to reveal a different picture of autism (8). New computational techniques are emerging to complement the observational instruments. Indeed, they promise to help advance diagnosis and screening tools to a new generation of a more dynamic, objective, quantitative science of autism, a science that would help uncover the readiness potential for social, emotional, and communication exchange (8, 21).

The new digital tools reach beyond the limits of the naked eye and can get to a micro-level of social and emotional nuances critical for conveying gestural communication and building rapport and trust among humans (22, 23). Bringing awareness of such nuances could then make them more accessible to diagnosticians and significantly improve the current clinical methods. Examining people through a new digital lens may shed new light on the social and emotional competencies of autistic individuals. What if we have been wrong all along and, in fact, autistic individuals do have empathy, do have emotions, and do want social interactions, but we cannot see it through the traditional lens that relies exclusively on visual observation?

Quantitative biometrics are beginning to become more ubiquitous in our behavioral sciences. They include methods based on eye tracking (24, 25), facial expressions (26), reaching movements (22, 27–29), gait (30), neural correlates from Electroencephalography (EEG) (31–33), Magnetoencephalography (MEG) (34), and functional Magnetic Resonance Imaging (fMRI) resting-state motion (35–39), among others. Nevertheless, most of this basic research heavily depends on instrumentation that is much too invasive for autistic individuals who suffer from hyper- and hypo-sensitivity to touch (40), movement (41–48), temperature-pain (49–51), and self-generated involuntary motions (39) that tend to keep the autonomic systems highly dysregulated (52, 53).

New non-intrusive data acquisition techniques amenable to capturing biological motions embedded as nuance in our behaviors are now becoming available thanks to recent advances in computer vision (54, 55). Among them are facial recognition and facial tracking methods that can extract behavioral states from video data (54, 55). They hold promise to identify differences between autistic individuals and controls (24, 26, 56). The few methods that exist are nevertheless still too taxing on autistic individuals. They may require a long time to acquire the data, which requires focus—a challenge for some autistic individuals. They may also heavily rely on heuristics and manual setting of threshold values required to reduce and analyze the large amounts of data that lengthy assays produce.

In this work, we combine a brief assay requiring merely 5 seconds per task to acquire video data in non-intrusive ways using commonly available means such as a webcam, an iPhone, or a tablet. We combine this brief data acquisition assay with new analytics that maximize data extraction and permit a direct personalized assessment of the micro-movements of the face as a person makes facial gestures on command. We seek to identify commonalities across the autistic and neurotypical facial coding ranges at a micro-level to characterize facial nuances beneath awareness that could nevertheless play a key role in building social rapport between autistic individuals and neurotypicals. We posit that by bringing those nuances to awareness, both groups could better co-adapt and coexist while minimizing judgmental opinions and rather presuming that competencies do exist across the human spectrum at a level that we can now make accessible to share.

## 2 Materials and methods

### 2.1 Participants

The data set comprised individuals on the autism spectrum and typically developing or typically developed controls (TDs). The broad spectrum included non-speakers with mid to high support needs (ASD-HS) who have a diagnosis of ASD and within this group a subgroup with a diagnosis of apraxia. The ASD group also comprised individuals with low support needs (ASD-LS) who can communicate through spoken language. Among the TDs are also adults who are parents of the non-speaker ASD-HS participants and of those with apraxia. Some of the moms reported diagnoses of acquired autoimmune disorders, depression, bipolar disorder, and attention deficit hyperactivity disorder (ADHD), which we have generally grouped under TD MomM. Other moms reported healthy aging (denoted TD Mom). TD dads did not report any neuropsychiatric disorders. **Table 1** reports on the various participating sites. These included a school of children with special needs, two clinics, and one social event.

**Table 1:** Participants recruitment and demographics.

Location	N	Participant type	Age range	Task assays
School 1	29	ASD (LS)	6–10	Resting, surprised, happy, baseline, pre and post OT, and SLP therapies
Social event	9	ASD (HS)	9–21	Anger, happy, sad, surprise, resting
Social event	9	ASD (HS)—apraxia	14–21	Anger, happy, sad, surprise, resting
Social event	11	TD-Mom	50–60	Anger, happy, sad, surprise, resting
Social event	8	TD-MomM	50–60	Anger, happy, sad, surprise, resting
Social event	10	TD-Dad	50–75	Anger, happy, sad, surprise, resting
Clinic-spa	20	TD	20–50	Anger, happy, sad, surprise, resting, pre and post Feldenkrais therapy
School 2	12	TD	7–29	Anger, contempt, disgust, fear, happiness, sadness, surprise
School 3	9	ASD (LS)	7–12	Anger, contempt, disgust, fear, happiness, sadness, surprise

Demographics: Total of 126 participants, 70 TD (8 with acquired neuropsychiatric disorders, 32 healthy, and 9 with self-reported autoimmune disorders) and 56 ASD (29 speakers with low support needs, 18 non-speakers with high support needs, and 9 non-speakers with high support needs and a diagnosis of apraxia).

ASD, autism spectrum disorder; LS, low support; HS, high support; SLP, Speeech Language Pathologist; TD, typically developed.

#### 2.1.1 Strategies for recruiting participants

Participants were recruited through word of mouth, using Institutional Review Board (IRB)-approved flyers distributed across the various Rutgers University campuses. Furthermore, non-profit organizations were contacted, and the IRB-approved flyers were distributed at conferences and various events, schools, and clinics. The Principal Investigator (PI) and students traveled to various events, schools, and clinics to record participants. Clinicians and school principals distributed IRB-approved letters to parents to further assist in student enrollment. Those who expressed interest in participating in the study were recruited.

### 2.2 Data acquisition and processing

We acquired the data using video cameras embedded in iPhones, tablets, or webcams. We also used a research app that facilitates doing so at home, clinics, or schools without having to come to our lab (see picture in **Supplementary Figure 1A**). Upon IRB consent, the app guided the person to a session of practice (5 seconds), then resting (5 seconds), and then a series of micro-expressions from instructed emotional gestures, each also lasting 5 seconds. In some participants, we only used smiling (as in happy) and opening of the mouth (as in surprised) (see picture in **Supplementary Figure 1B**). In other participants, we used those micro-expressions and other emotional gestures such as anger, disgust, contempt, fear, and sadness. The latter combined with the former formed seven facial micro-expressions popularized by Paul Eckman (57) and other researchers. We used the instructions by Vanessa Van Edwards (see Note 1) to provide guidance (to the caregiver) on how to produce motions of a facial expression to instruct the participant. In some cases, we directly instructed the participant.

**Figure 2:**
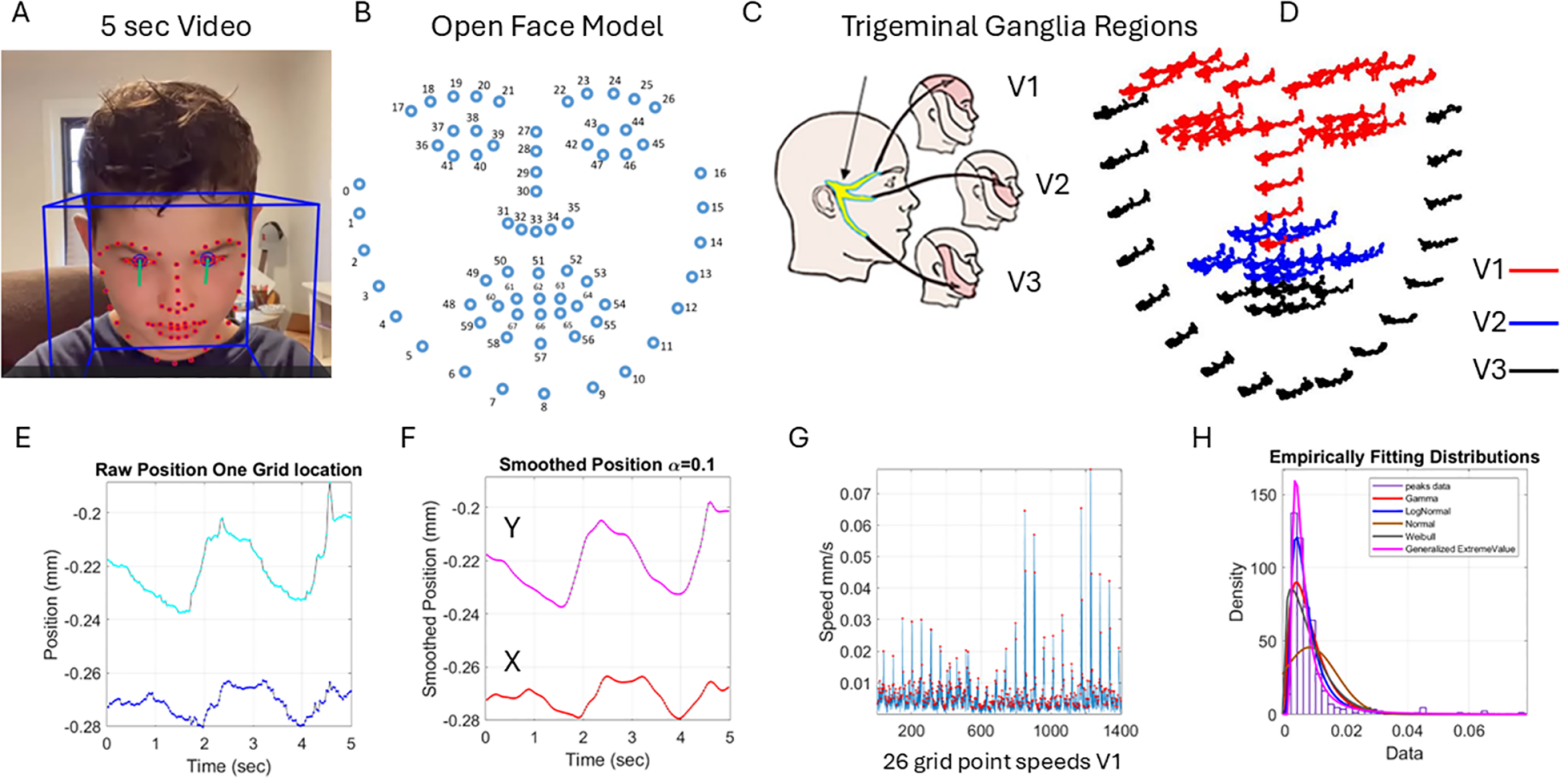
Pipeline of data acquisition and pre-processing. **(A)** Sample grid from OpenFace was extracted from 5 seconds of video data captured using a research app. **(B)** OpenFace grid was used to track the 68 markers numbered from 0 to 67. **(C)** The trigeminal ganglion-inspired parcellation of the face grid points into three facial subregions of V1, V2, and V3 to analyze the data according to subregions. **(D)** The 5-second positional pixel trajectories across the subregions. **(E)** One grid point 5-second positional trajectories of the X and Y coordinates converted from pixels to mm. **(F)** The smoothed position to enable proper differentiation to obtain the velocity with scalar values (speed) in panel G obtained by gluing all segments of the region (V1 in this case) and **(H)** obtaining the frequency histogram of the peaks (red dots) in panel **(G)** Multiple fitting distributions were examined and the continuous Gamma family of probability distributions selected as the best fit in a maximum likelihood sense. Written informed parental consent was obtained from the parents of the individual.

The purpose of the experiment was not to identify emotions but rather to capture micro-movements of the facial action units (AUs) and facial grid points that we can extract from the videos using the OpenFace software (54). **Figure 1B** shows examples of the grid that we can extract from brief videos in different populations (neurotypical, ASD-HS, and ASD-HS with the additional diagnosis of apraxia). Importantly, we can see the motion trajectories of the pixels’ positions as they change during resting *vs.* other states of smiling or surprise. **Figure 2** explains the pipeline of data parsing. Upon collecting the videos, we ran OpenFace (see Note 2, https://github.com/TadasBaltrusaitis/OpenFace/wiki) and retained the facial grid and AUs (see **Supplementary Figure 2** and Note 2). We then used the trigeminal nerve (cranial nerve V) regions shown in **Figure 2C** to parse out three subregions of the face (ophthalmic, maxillary, and mandibular), which we named V1, V2, and V3, respectively. These were colored differently to map those grid points of **Figure 2B** in correspondence with the regions in **Figure 2D** innervated by the trigeminal nerves. These are important for sensing movement reafference throughout key facial regions that enable social and emotional communication (e.g., the eyes, ears, mouth, lips, tongue, and mandibular regions).

The 5 seconds of the assay during the resting state is shown in **Figure 2E** for the positional X and Y pixel coordinates (normalized to adjust for discrepancies in distance from the face to the camera). Please see the subsection below. We smoothed out the facial grid points’ trajectories using in-home developed software that employs the spline interpolation toolbox MATLAB version R2023b. We then obtained the speed quantities by proper differentiation and glued all segment trajectories in each point of the grid corresponding to the subregions spanned by 68 points on the grid of **Figure 2B**. These are 26 points of the grid in **Figure 2B**, V1 (17:30 33 36-47); 17 points of the grid, V2 (31 32 34 35 48 49:53 54 68:64); and 25 points of the grid, V3 (0:16 55:59 65:67).

**Figure 2G** shows the speed points corresponding to the speed segments of the 26 grid points of V1. We focused on the peaks (marked as red dots in **Figure 2G**) to obtain the frequency histogram of their distribution across all these points of the grid during the 5-second task. This is shown in panel **Figure 2H**. We were not interested in the temporal component at this point, and for that reason, we did not treat this sequence as a time series but rather focused on the set of variations in the amplitude of the speed signal. Shuffling the points in each region to glue them in a different order did not impact the shape of the histogram. The overall result of the work remained, and the analysis produced similar parameter ranges. However, for consistency, to glue the speed segments, we set a fixed order of the points in the grid and used that order for the entire data set. This order is as explained above in V1, V2, and V3 based on the proximity of the grid points because of inherent synergies and co-dependencies of their motions. These co-dependencies are influenced by allometric effects of the distance between points, with lengths that vary across the population due to anatomical and size differences.

A sampling at 30 Hz and 5 seconds worth of data gave us 150 frames per grid point for a total of 3,900, 2,550, and 3,750 frames for V1, V2, and V3, respectively. These in turn provided over 100 peaks of the speed amplitude, such that empirical estimation of the stochastic signatures of speed variability provided enough statistical power and tight 95% confidence intervals. We caution that if the analysis were to focus on the temporal dynamics of the time series data, one would need to double the lower bound of data time to 10 seconds and focus on the individual time series. For other types of temporal analysis, one can use the series of peak width (ms) and analyze the stochastic signatures of the glued data. However, here, we focus on the speed amplitude variations rather than on the temporal information. These are different aspects of the problem, but the methods provided here for analyses of the variations in speed amplitude can also be used for temporal dynamics-related data (see, for example, 30, 58).

#### 2.2.1 Distance normalization

To account for the translation of the subject along the optical axis, we performed the following z-score normalization: for each frame, we took the mean and standard deviation of each positional coordinate component, x and y, and performed a transformation like a z-score calculation:


$$z_{x}=\frac{\left(x-\mu_{x}\right)}{\sigma_{x}},\;z_{y}=\frac{\left(y-\mu_{y}\right)}{\sigma_{y}}.$$

This transformation thereby produces a time series of normalized faces (collections of 68 points), each with a mean of 0 and a standard deviation of 1. While this normalization does not prevent distortions from head rotations or lens compression, it nonetheless is an essential preprocessing step to create invariance to the distance from the camera.

#### 2.2.2 Micro-movement spikes and distribution fitting

The distribution of the peaks’ amplitude is subject to distribution fitting, yielding the continuous Gamma family of probability distributions as a good fit to capture both the autistic and neurotypical stochastic ranges. This is so because the autistic sample also includes the memoryless exponential distribution, which has a shape parameter a=1 in the Gamma family.

**Figure 3:**
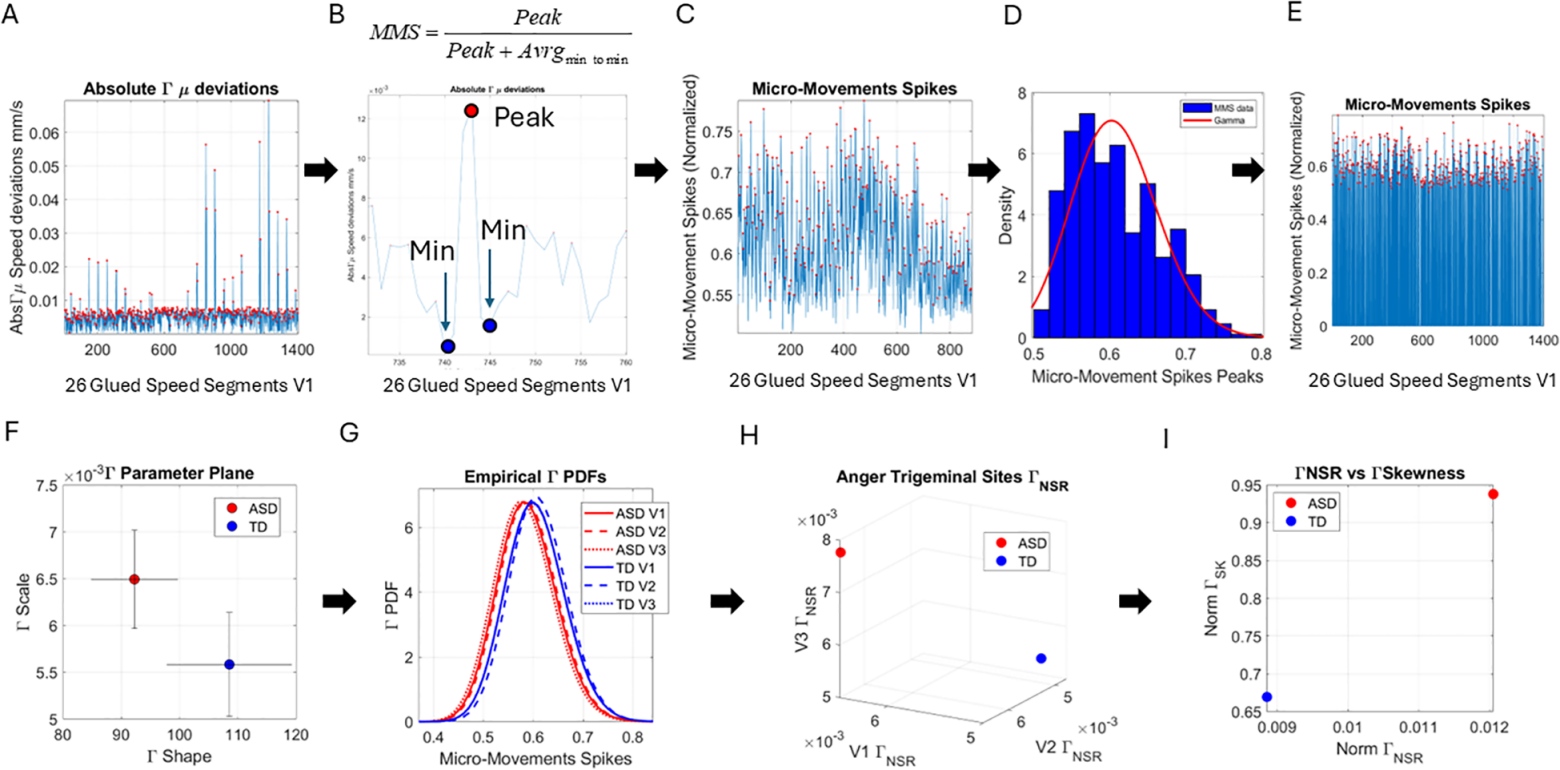
Pipeline of data analysis. **(A)** Absolute deviations from the empirically estimated Gamma mean ( $\Gamma_{\mu }$ ) were obtained for each point of the set, and the peaks were extracted for local scaling. **(B)** Local scaling to normalize the speed amplitude and dampen effects of anatomical differences and lengths across faces. The local peak was scaled by the sum of the peak value and the average value of its neighboring local minima. **(C)** The micro-movement spikes (MMSs), considering only the frames with the speed deviation peaks above 0 value. **(D)** The histogram of the MMSs and the fitted Gamma probability distribution function. **(E)** The full MMSs inclusive of 0-valued entries in **(A)** retaining all original frames distributed bimodally between small and larger deviations and can be used in other analyses beyond the scope of this paper. **(F)** The Gamma parameter plane spanned by the shape and the scale values and 2 representative values derived from the empirical speed’s MMSs with 95% confidence intervals. **(G)** Empirical Gamma PDFs corresponding to the representative values in panels **(F, H)** Parameter space spanned by the Gamma scale (NSR) obtained from all points in V1, V2, and V3, also for the representative participants of panels **(F, I)** Parameter plane spanned by the norm of the V1, V2, and V3 Gamma NSR and the norm of the V1, V2, and V3 Gamma Skewness from the example in panel **(F)**.

We then empirically estimated the Gamma mean in each subregion $\Gamma_{\mu }=a\cdot b$ , and for each point on the ordered series of grid points in the subregion, we obtained the absolute deviations from the empirical $\Gamma_{\mu }$ . This is shown in **Figure 3A** with the peaks of the absolute deviations from the $\Gamma_{\mu }$ marked red dots. For each peak, we normalized the value using the formula in **Figure 3B** to scale out neighboring effects related to anatomical differences and disparities in distances between the grid points across the participants (59). **Figure 3C** then shows the data from the normalized peaks, which we named micro-movement spikes [granted US patents (60–63) filed in 2012] and for which we obtained the frequency histogram and Gamma fitting. These are also expressed in **Figure 3E** as spikes ranging in the real valued interval from 0 to 1 across the original number of points in the series of **Figure 3A**. Compare these to **Figure 3C** with fewer points of non-zero absolute deviations from the empirical Gamma mean of the region (V1 in this case). The micro-movement spike (MMS) comprising all frames can be turned into binary spikes and other sets of information theoretical tools employed (as in recent work from our lab; 64) when we used time series data of a longer time range. In the present work, since we deliberately chose the lowest possible time range (5 seconds) that still enables statistical power, we focused on stochastic analyses instead.

Upon estimation of the best fitting distribution to the peaks of the non-zero deviations from the empirical $\Gamma_{\mu }$ , we found once again that the continuous Gamma family of probability distributions best fits the data (in a maximum likelihood estimation sense). We then plotted the Gamma shape (*a*) and the Gamma scale (*b*) parameters of the Gamma family thus obtained on a parameter plane with 95% confidence intervals. In **Figure 3F**, we show an example of an ASD *vs.* a TD participant, and in **Figure 3G**, we show the empirical Gamma Probability Density Function (PDFs) for each of the three facial regions, V1, V2, and V3, that we have defined in this study. **Figure 3H** shows another parameter space spanned by the Gamma scale (the noise-to-signal ratio $\Gamma_{NSR}=\frac{\Gamma_{\sigma }}{\Gamma_{\mu }}=\frac{a\cdot b^{2}}{a\cdot b}=b$ ) of each subregion. We then used the scalar value of the vectorial representation of these respective noise levels (V1, V2, and V3), i.e., the Gamma Noise to Signal Ratio (NSR) and the Gamma skewness of each of the empirical distributions, to plot the scalar quantities as points on a parameter plane. This parameter plane spanned by these two dimensions of the data is shown in **Figure 3I** for the example of ASD and TD representative participants.

Lastly, we measured, pairwise, the distances between the distributions of the MMS peaks for each face region and participant. To that end, we used the Earth Mover’s Distance (EMD) (65, 66) and plotted the color maps for each of the facial gestural assays. We used the resulting matrices as input to a tree classifier, and given the number of subtypes of interest, we then quantified the percentage of ASD *vs.* TD participants in each cluster that the algorithm found. These were reported on each leaf of the branches denoting emerging subtypes in the tree structure.

### 2.3 Action unit identification

The OpenFace interface reveals universal AUs present (as a binary matrix of N frames by 18 columns) in each facial expression. This is in the form of an N × 18 matrix, where N is the number of frames collected at 30 Hz and 18 are AUs defined in the OpenFace GitHub site (see Note 2). The intensity of the AUs is also defined in an N × 17 matrix.

Across frames, we used the edit distance (67) of the column-wise binary vector denoting the presence of the AU to measure its frequency across the 5 seconds of motion yielding approximately 150 frames per point in the grid (plus/minus two frames upon OpenFace estimation). Then, we obtained the most common pattern present (the mode) to denote the presence of the AU. This pattern was saved along with its corresponding intensity values. We saved intensities equal to and above the 0.001 level. We did this for the individuals of the ASD and TD control groups. We then pooled across the intensity values and thus identified and reported on the frequency histograms of intensity values for the AUs that are present in each group.

## 3 Results

### 3.1 Stochastic differences between ASD and TD are captured at rest

**Figure 4:**
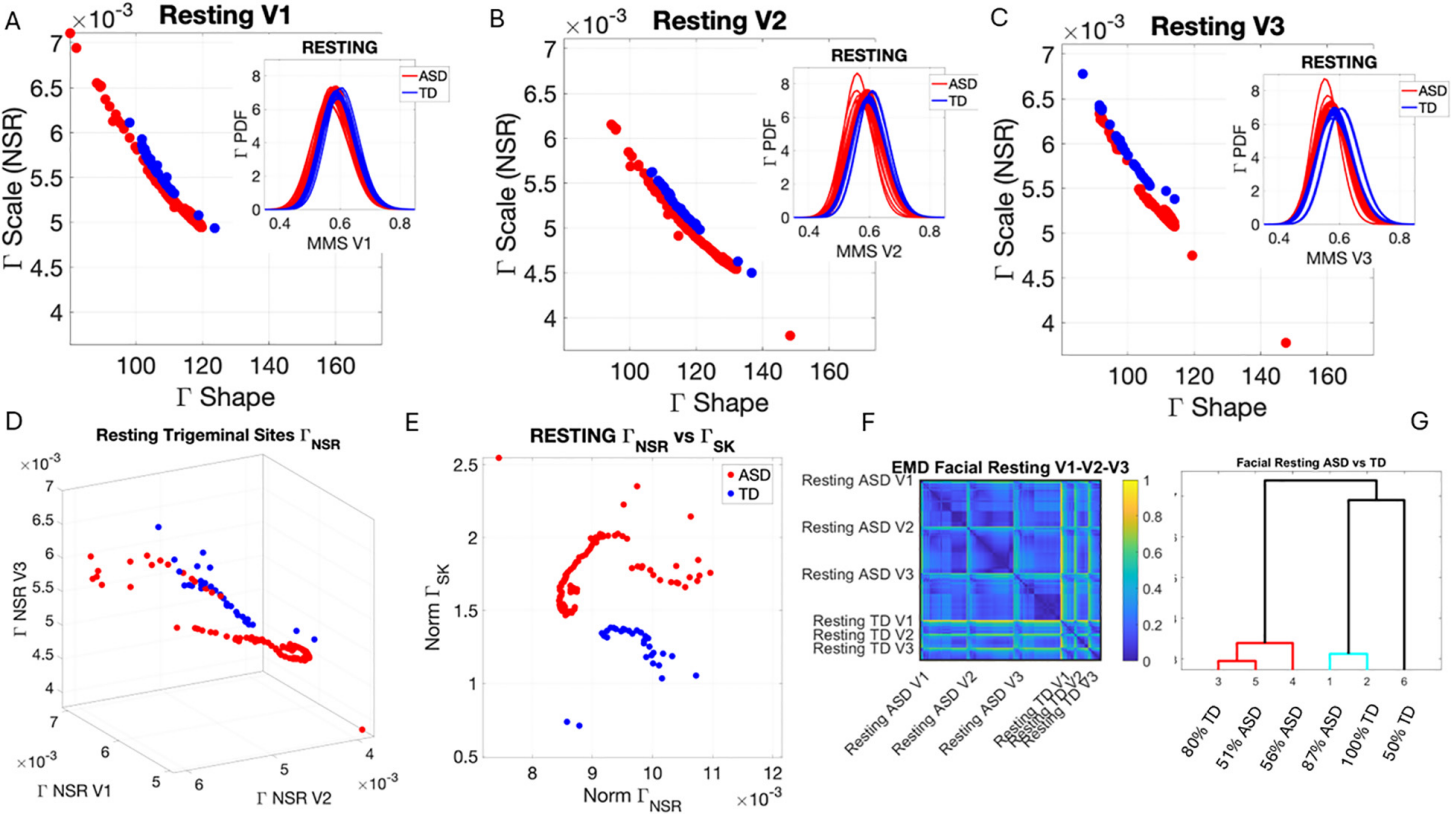
Results from the analysis of resting state data, whereby 5 seconds of the face at rest was captured on video, and the pipelines in **Figures 2** and **3** were executed. **(A–C)** Gamma plane results for each of the V1, V2, and V3 facial regions. Inset corresponds to the empirical Gamma PDFs showing shifted density and differences in dispersion and skewness. Notice the span of the ASD scatter with broader variability (along both dimensions) at rest than that of the TD controls. **(D)** Gamma NSR parameter space also reveals differences in the scatter between ASD and TD groups, with higher noise for the mandibular region V3 in ASD-HS apraxia subgroup. Lower skewness in TD is also evident. Higher skewness in ASD points at heavier tails in the empirical Gamma PDFs with higher speed deviations from the empirical Gamma (MMS speed peaks) mean. These represent rare events relative to controls with lower skewness tendencies. **(E)** Parameter plane spanned by the norm of the Gamma distributions' NSR (scale parameter) from the (V1, V2, V3) vector and the corresponding norm of the Gamma distributions' skewness of V1, V2, V3). (F) Color map of the EMD taken pairwise for ASD and TD participants across all regions V1, V2, V3. (G) Corresponding tree with cluster composition. **(F)** Pairwise EMD obtained for both groups, and each of the face subregions separate ASD from TD and provide structure denoting differences in distributions of the normalized speed peaks at rest. **(G)** Tree clustering of **(F)** outputs with different compositions in the 6 subtypes (3 face regions × 2 groups). ASD, autism spectrum disorder; TD, typically developed; HS, high support; EMD, Earth Mover’s Distance.

At rest, TD individuals produced separable patterns of facial micro-movements from ASD participants. Each one of the facial regions showed separable patterns, expressing more variability in the ASD participants across all subregions. **Figures 4A–C** show the patterns of Gamma shape and scale parameters along with the empirical PDFs that shift in ASD relative to controls, with the standardized absolute deviations from the Gamma mean corresponding to the micro-movements’ spikes of the facial subregion.

Along the dimensions of the Gamma NSR in **Figure 4D**, we observed orderly patterns, suggesting that this projection of the high-dimensional data may produce an embedding of contiguous points distinguishable between ASD and TD. These are conserved in **Figure 4E** along the parameter plane that focuses on the scalar quantities. In **Figure 4D**, elevated NSR in V3 for a subset of participants on the spectrum revealed correspondence with those with the ASD-HS subset with the diagnosis of apraxia. These patterns expressed statistically significant differences in the Gamma scale (NSR) and higher skewness parameter (indicative of heavy-tailed distributions with more frequent appearance of high-speed motions in the MMSs). Please see **Supplementary Figure 3** showing pairwise Wilcoxon rank-sum test statistics (equivalent to the Mann–Whitney U-test) for ASD *vs*. TD inclusive of micro-expressions during the resting state *vs*. micro-expressions for anger, happiness, sadness, and surprise. Area V1 was consistently significantly different between TD and ASD participants across the resting state and all other micro-expressions (taken pairwise, p < 0.01). Furthermore, in V1, within each of the cohorts, the resting state micro-expressions were significantly different from those in all other states. In the ASD group, happiness and surprise also differed significantly from all other micro-expressions (p < 0.01). In the TD group, anger *vs*. surprise was significantly different at p < 0.01, and sadness *vs*. anger was significantly different at p < 0.05. In areas V2 and V3, statistical differences followed more complex patterns.

In V2, TD at resting state differed from all ASD micro-expressions but only significantly for resting and happiness at p < 0.01. TD anger and surprised differed significantly from ASD happiness at p < 0.01. Within the TD group, resting differed from happiness and sadness, as did happiness and sadness relative to surprise, all at p < 0.05. Within the ASD cohort, anger differed from happiness and happiness from anger at p < 0.01. Surprise in ASD differed from all micro-expressions with significance relative to rest and happiness, p < 0.01.

In V3, the resting micro-expressions in ASD differed from all micro-expressions in TD (p < 0.01). ASD happiness differed significantly from TD anger and TD happiness (p < 0.01). Within the ASD cohort, ASD happiness differed from resting, anger, sadness, and surprise (p < 0.01). ASD surprise differed from all micro-expressions with significance at p < 0.01 for happiness and p < 0.05 for resting, while anger and sadness did not reach significance relative to surprise.

In **Figure 4D**, we compare the Gamma NSR along each of the facial subregions. The patterns separate participants with ASD-HS apraxia along the V3 (mandibular region) from other ASD participants and TD controls. All ASD distributions from the resting condition showed higher skewness than those from the TD controls and broader ranges of the Gamma NSR, with heavier right-tailed distributions for the ASD-HS participants. This can be appreciated in **Figure 4E**. Furthermore, there is a visible structure in the EMD matrix of **Figure 4F** denoting the pairwise differences in distributions according to the similarity metric (EMD) across the participant’s three facial subregions. Within the TD group, we saw similarities in the distributions of standardized speed MMSs in the lower values of the EMD. Furthermore, clear differences between the ASD and TD groups were captured with automatic clustering using six groups (ASD *vs.* TD and three face regions) that break down the composition of each subtype in **Figure 4G**.

### 3.2 Facial micro-movements during expressions of anger, happiness, sadness, and surprise show significant stochastic differences between ASD and TD

**Figure 5:**
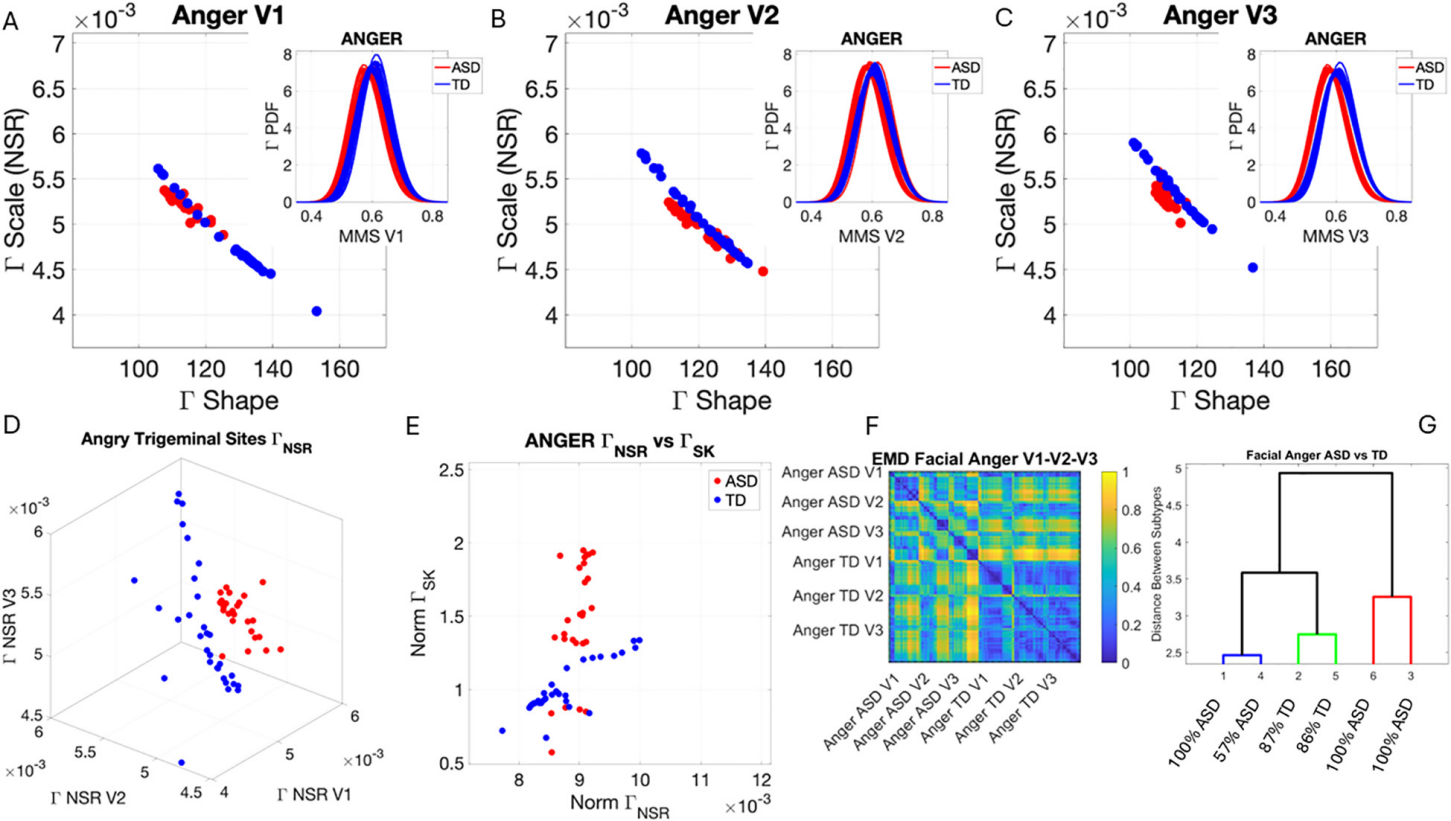
Results from the analysis of anger micro-expressions, whereby 5 seconds of the face was captured on video upon instruction of how to enact facial micro-expressions of an angry face. The pipelines in **Figures 2** and **3** were executed to appreciate the shifts from resting state in **Figure 4**. **(A–C)** Scatters on the Gamma plane show broader ranges of motion in TD signaling more variability in the two dimensions across the TD population. **(D)** The ASD scatter on the Gamma NSR space separates from the TD scatter, which spans broader ranges of noise (variability in speed MMSs) across all facial subregions. **(E)** Higher skewness scalar quantities in ASD signal heavier-tailed empirical Gamma PDFs during anger expressions, denoting more rare events of faster deviation peaks from the empirical Gamma mean. **(F)** Pairwise EMD metric shows more similarity among controls (blue tones with low EMD values) than ASD and differences in normalized speed deviations (MMSs) across all regions. ASD group is more similar in V1 (comprising the eyebrow and eye micro-expressions). **(G)** Different clusters and their composition for the 6 subgroups (2 groups × 3 subregions of the face). TD, typically developed; ASD, autism spectrum disorder; MMSs, micro-movement spikes; EMD, Earth Mover’s Distance.

**Figure 6:**
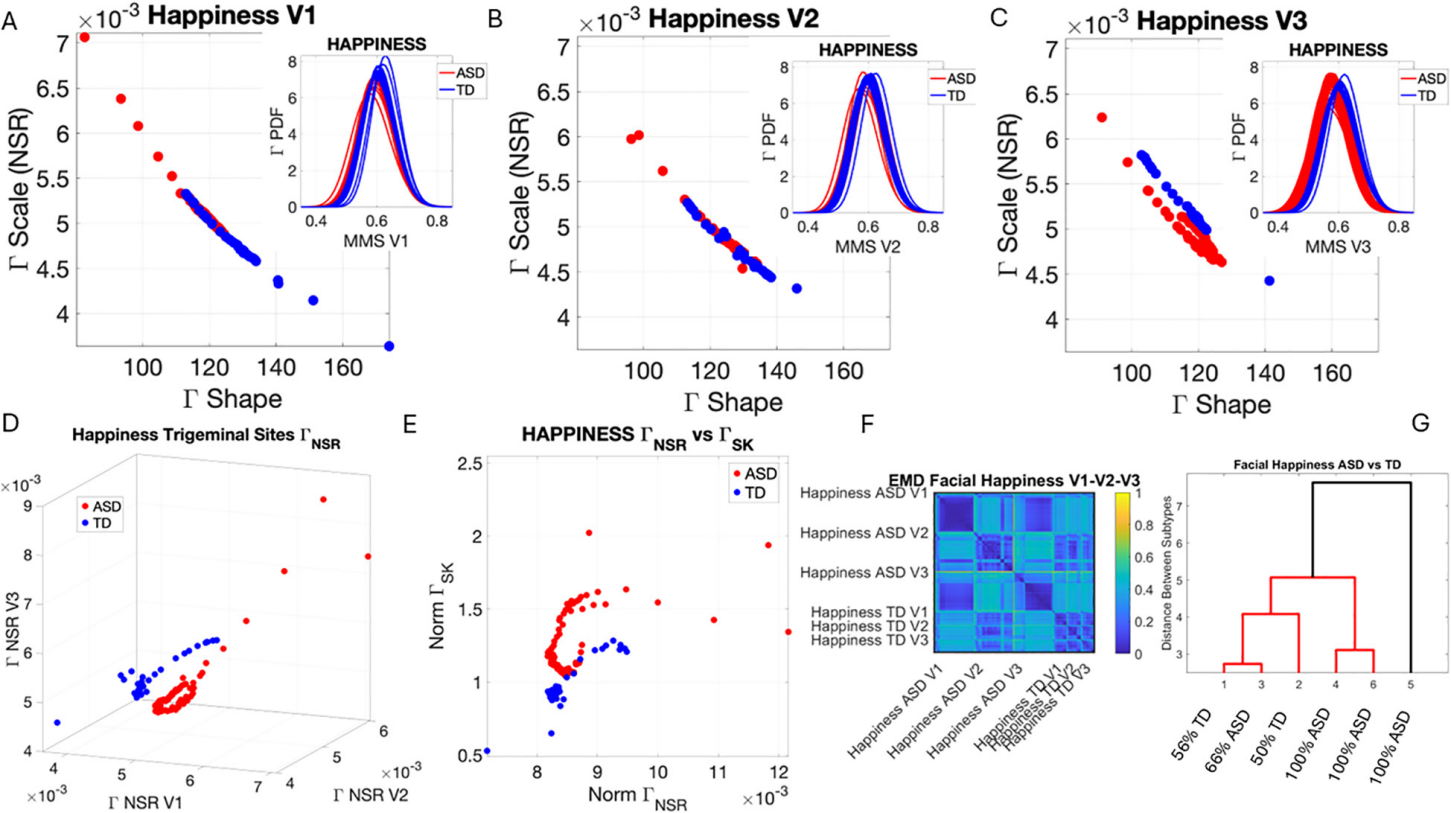
Results from the analysis of happiness micro-expressions, whereby 5 seconds of the face was captured on video upon instruction of how to make a happy face by smiling. The pipelines in **Figures 2** and **3** were executed to appreciate the shifts from resting state in **Figure 4**. **(A–C)** Gamma plane scatters show marked differences in the V3 mandibular area engaged in motions to produce a smile. Subregions V1 and V2 show more overlapping patterns, apart from outliers with higher noise, which correspond to the ASD-HS apraxia subgroup. **(D)** Separation between the scatters of TD and ASD show higher ranges of speed MMS variability in TD motions and outliers (ASD-HS apraxia subgroup) span broader ranges of noise. **(E)** The ASD scatter shows higher Gamma skewness ranges indicating heavier right tails of the distributions as with resting and anger, with more rare events (higher deviations from the Gamma speed mean boasting faster changes in position of the points). **(E)** Parameter plane spanned by the norms of the Gamma probability distributions' NSR and skewness from (V1, V2, V3) showing the higher skewness values for ASD (denoting the accumulation of more rare events on the right tail (higher speed fluctuations away from the empirical Gamma mean) (F) Color map from pairwise EMD across ASD and TD face regions V1, V2, V3. (G) Corresponding tree cluster with compositions from each group. **(F)** Pairwise EMD shows similarity within V1, most of the groups in V2 and V3 in ASD across the group, and differences in V1–V2 and V2–V3 comparisons with ASD. The TD group boasts more similarity across regions. Pairwise comparisons show V2 as the most similar region between TD and ASD. ASD, autism spectrum disorder; HS, high support; TD, typically developed; MMS, micro-movement spike; EMD, Earth Mover’s Distance.

**Figure 7:**
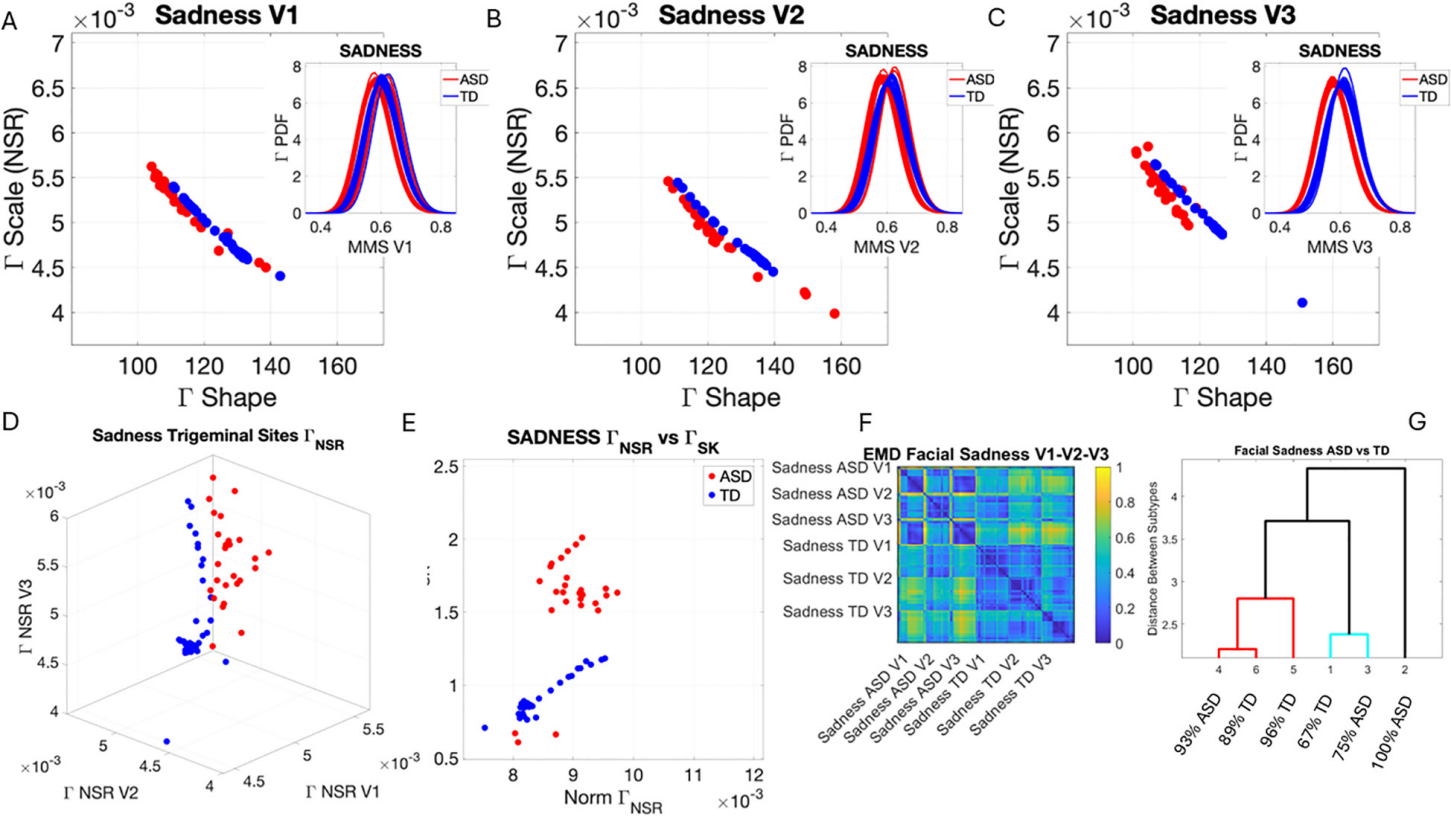
Results from the analysis of sad micro-expressions, whereby 5 seconds of the face was captured on video upon instruction of how to make a sad face (as in Note 1). The pipelines in **Figures 2** and **3** were executed to appreciate the shifts from resting state in **Figure 4**. **(A–C)** Gamma plane scatters and insets showing the empirical Gamma PDFs. Notice the marked shifts in each case with V3 at the highest differentiation. **(D)** Both scatters on the Gamma NSR parameter space showing the departure of ASD from TD scatter. **(E)** The Gamma NSR scalar quantity *vs.* the Gamma skewness scalar quantity also separates the groups. **(F)** The pairwise EMD matrix showing differentiation within the ASD group and higher similarity within the TD group. Highly structured patterns of EMD across the three regions are marked between the two groups. **(G)** The tree clustering analyses and reporting on the composition of each subgroup. ASD, autism spectrum disorder; TD, typically developed; EMD, Earth Mover’s Distance.

**Figure 8:**
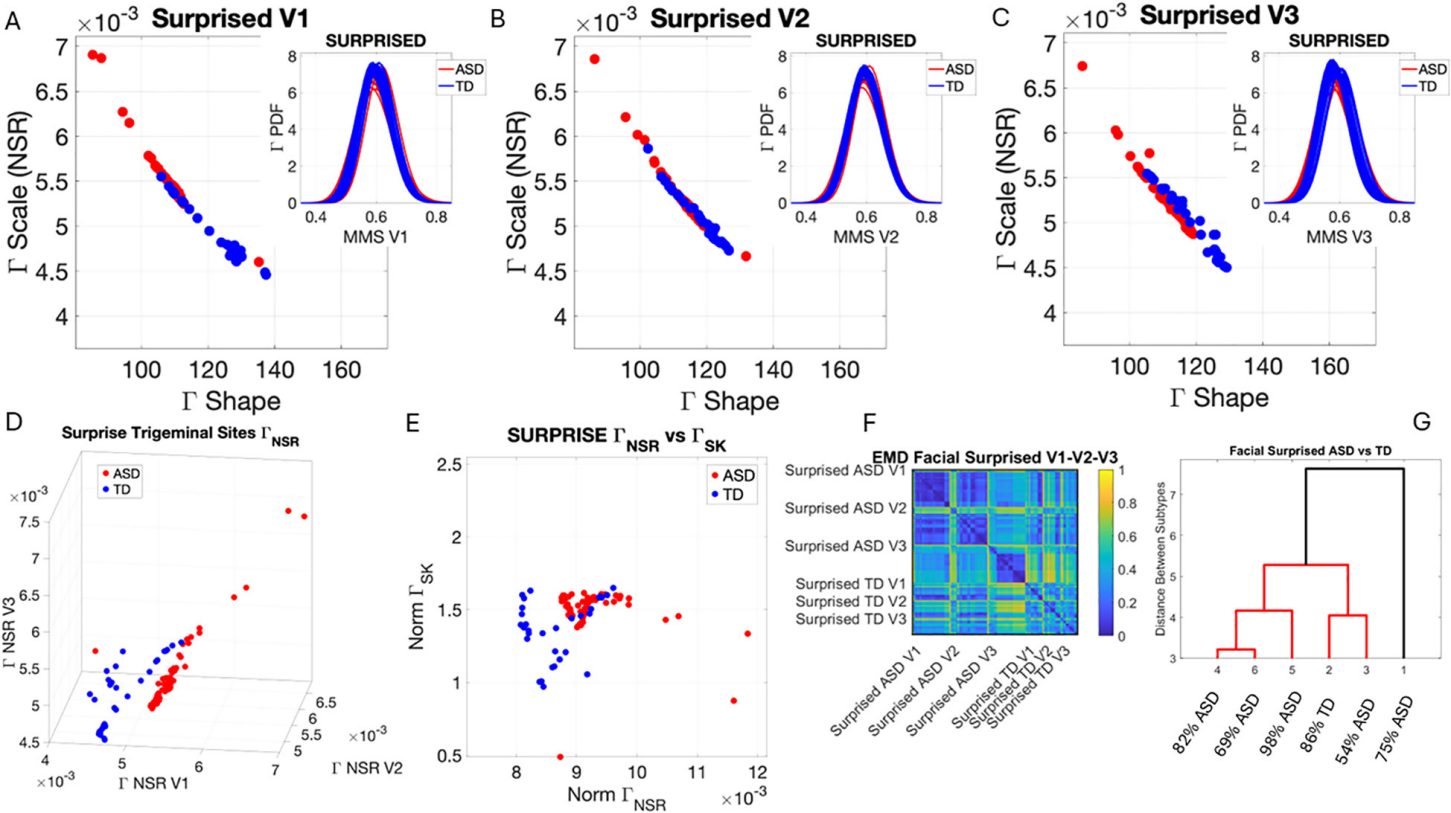
Results from the analysis of surprise micro-expressions, whereby 5 seconds of the face was captured on video upon instruction of how to make a surprised face (as in Note 1). The pipelines in **Figures 2** and **3** were executed to appreciate the shifts from resting state in **Figure 4**. **(A–C)** Gamma plane scatters with insets showing the corresponding empirically estimated Gamma PDFs. **(D)** Gamma NSR parameter space with separated groups. **(E)** Gamma NSR scalar quantity *vs.* skewness scalar quantity of the two groups. Notice the overlap with some of the members of the TD group along both axes and the separation of most TD participants along the NSR axis due to more uniformity in facial patterns across the TD group. **(F)** The pairwise EMD matrix showing structure within the ASD group indicative of V1–V2 similarity *vs.* V1–V3 differentiation. TD group also shows high structure within the group and highest departure in distributions from the ASD group for area V3. ASD, autism spectrum disorder; TD, typically developed; EMD, Earth Mover’s Distance.

In the subset of individuals where, upon resting, we measured anger, happiness, sadness, and surprise micro-expressions as instructed by the person taking the measurements (as in Note 2), profound differences were captured in the Gamma NSR (along all three facial subregions) and Gamma skewness by the empirical distributions’ shifts. **Figures 5**–**8** show these patterns for each of the parameter spaces of interest. Statistically significant differences were found across all pairwise comparisons using the rank-sum (Wilcoxon) test. For the scalar quantity of the Gamma scale, TD anger *vs.* ASD anger was significantly different at the alpha 0.05 level, p < 0.02, resting p < 8.4 × 10^−8^, sadness p < 5.7 × 10^−4^, surprised p < 7.57 × 10^−6^, and no significant differences in the $\Gamma_{NSR}$ micro-expressions for happiness between TD and ASD. The skewness scalar quantity also showed significant differences across all micro-expressions with p << 0.001.

The EMD matrices and automatic cluster analyses across these emotional gestures also revealed the composition of the different subtypes amenable to visualizing their differences. In all cases, the patterns clearly showed broad ranges of variability in the Gamma NSR of the ASD participants and higher skewness ranges for ASD. The latter corresponded to higher MMS speed peak deviations from the empirical Gamma mean of the group, estimated for the anger activity. Infrequent events of faster speed peaks in ASD than those in the TD distributions showed in the ASD group, thus resulting in higher skewness. The summaries of these parameter ranges are depicted in panels D and E of each of **Figures 5**-**8**. Likewise, the EMD matrices and tree clustering revealed the differentiation and inner similarities of the groups across the face subregions under study.

### 3.3 Action units reveal facial emotions in ASD at the speed micro-movement level

**Figure 9:**
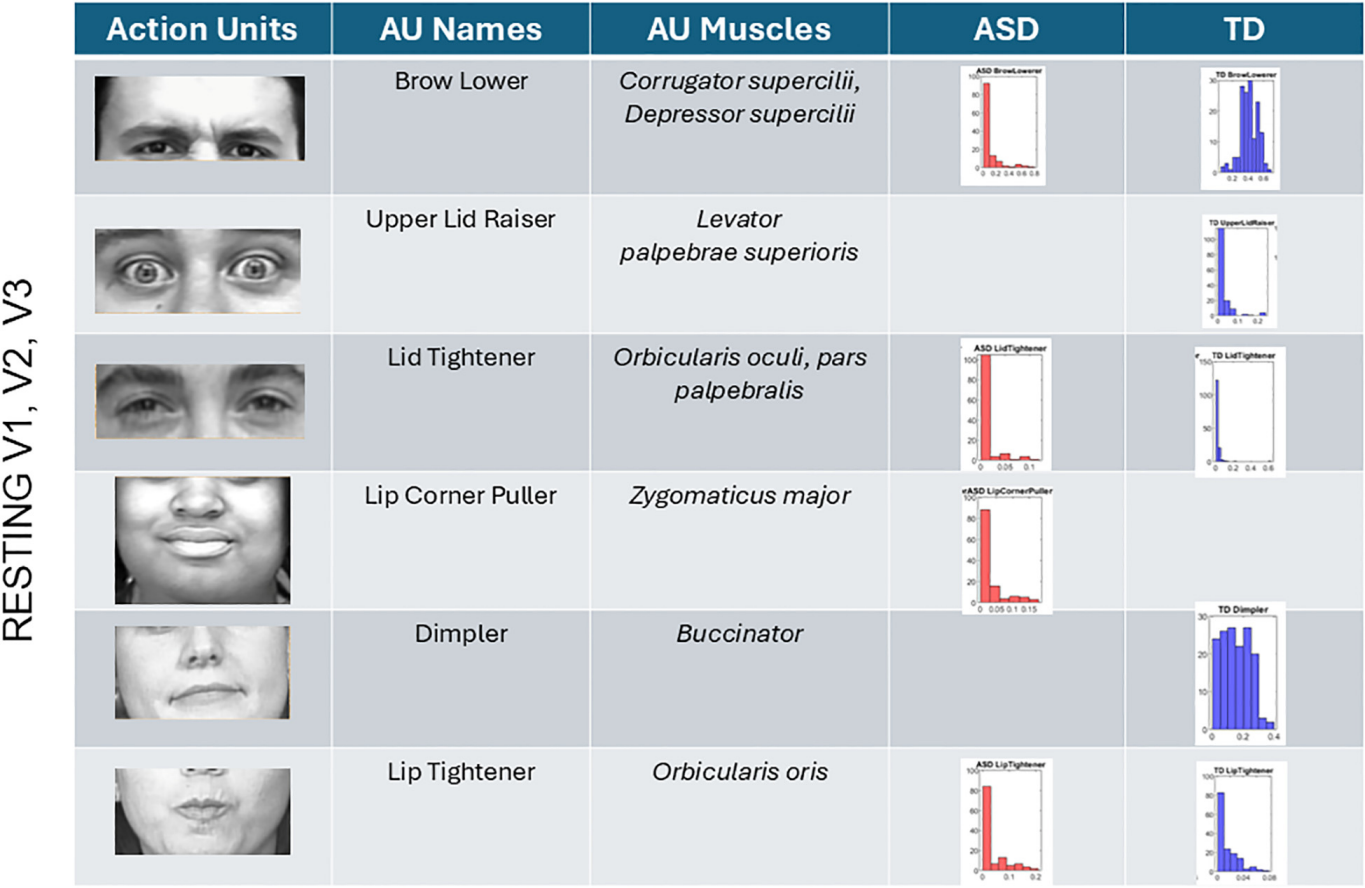
Action units (AUs) engaged during resting state. Overlapping AUs between ASD and TD participants in brow lower and lid tightener show different ranges of intensity but common presence in both groups. Upper lid raiser only appeared in the TD group. These are AUs corresponding to area V1. In V2 and V3 areas, lip tightener was commonly present in both groups albeit with different distributions of intensities. The AU lip corner puller was present in ASD but absent in TD, whereas the dimpler was present only in TDs. ASD, autism spectrum disorder; TD, typically developed.

The analysis of AUs revealed that most AUs present in TD participants are also present in ASD participants albeit with different distributions of intensities. At rest, TD did not show the lip corner puller present in ASD but showed upper lid raise absent in ASD participants. All other AUs present in TD were also present in ASD (brow lower, lid tightener, dimpler, and lip tightener). This can be seen in **Figure 9** for all AUs across the V1–V2–V3 facial subregions. There we also appreciate the differences in the intensity values and their distributions.

**Figure 10:**
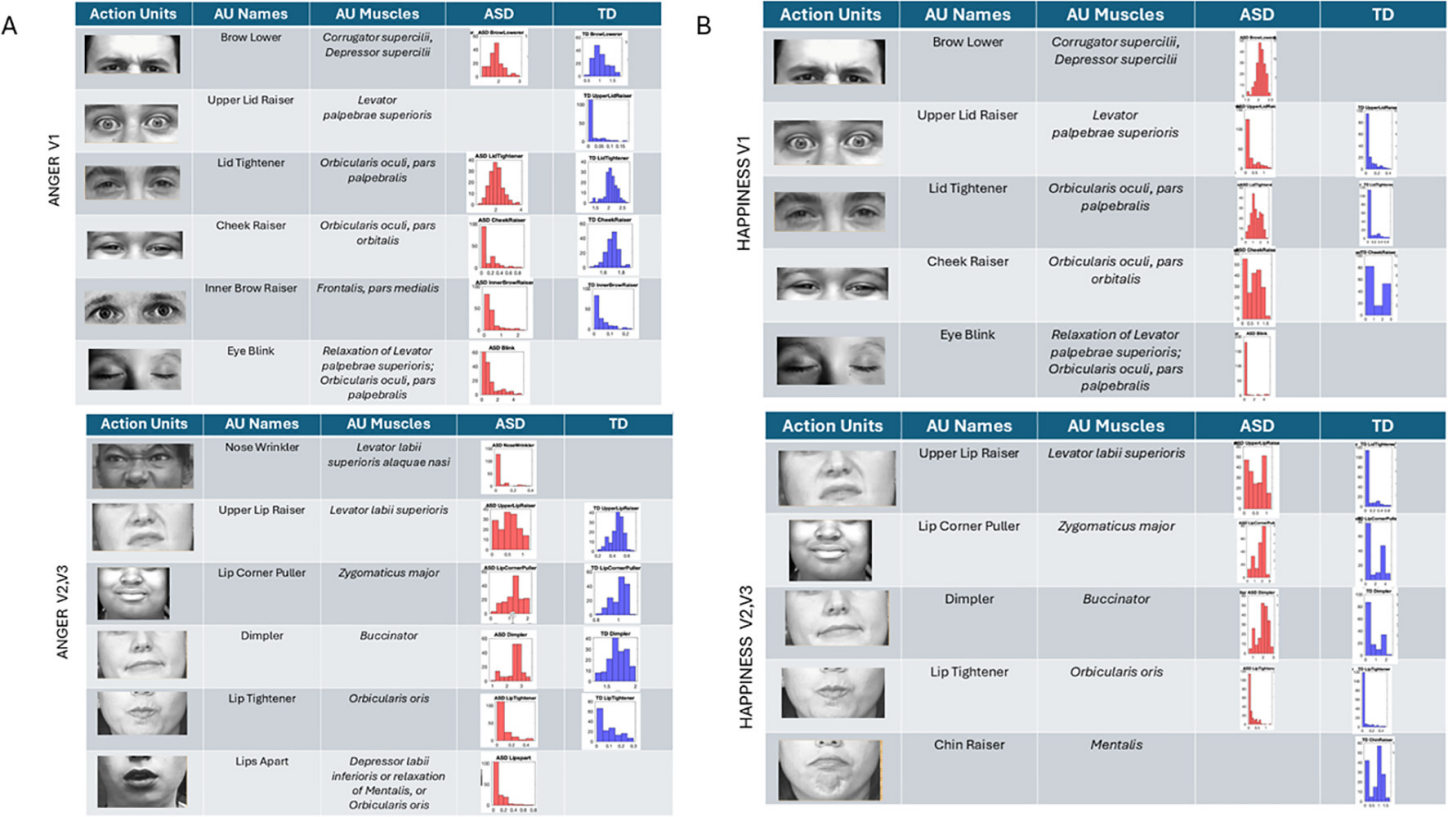
Action units for anger and happiness were also commonly present in both groups, but the ranges of intensities and their distributions differed between groups. The ASD group had AU eye blink present, but this was absent in the TD group for both **(A)** anger and **(B)** happiness. Upper lid raiser was absent in anger for ASD but present for TD. In happiness, brow lower (a common AU for anger) was present in ASD but absent in TD. ASD, autism spectrum disorder; AU, action unit; TD, typically developed.

Anger (**Figure 10A**) reveals the presence of the brow lower for both ASD and TD, with higher intensity across ASD participants and different intensity distributions relative to TDs. The TD group showed upper lid raiser, which was absent in the ASD group. Lid tightener was also more intense in ASD than TD and had a different distribution of intensities than TD. Cheek raiser was less intense in the ASD group than in the TD group, with significantly lower intensity range and values. Inner brow raiser was present in both ASD and TD but was far more intense in ASD than in TD. ASD had an eye blink AU present, which was absent in TD.

In subregions V2 and V3, anger speed motions from the facial expression revealed nose wrinkling and lips apart in ASD but not in TD. All other AUs were present in both groups, with differences in the range of intensities. Lip corner puller and lip tightener had comparable intensities in TD and ASD, but the dimpler AU was more intense in ASD than TD.

Happiness (**Figure 10B**) speed micro-motions revealed the presence of brow lower in ASD (commonly present in anger micro-expressions) but absence in TD. Upper lip raiser was present in both groups but expressed with higher intensity in ASD. Likewise, lid tightener was present in both but far more intense in ASD than TD, with fundamentally different distributions of intensities across the two groups’ participants. Cheek raiser, a prominent AU in micro-expression of happiness (see Note 1), was present in both groups but more intense and with broader ranges of values in TD than ASD. As with anger, blinking was present during happiness gestures in ASD but absent in TD.

**Figure 11:**
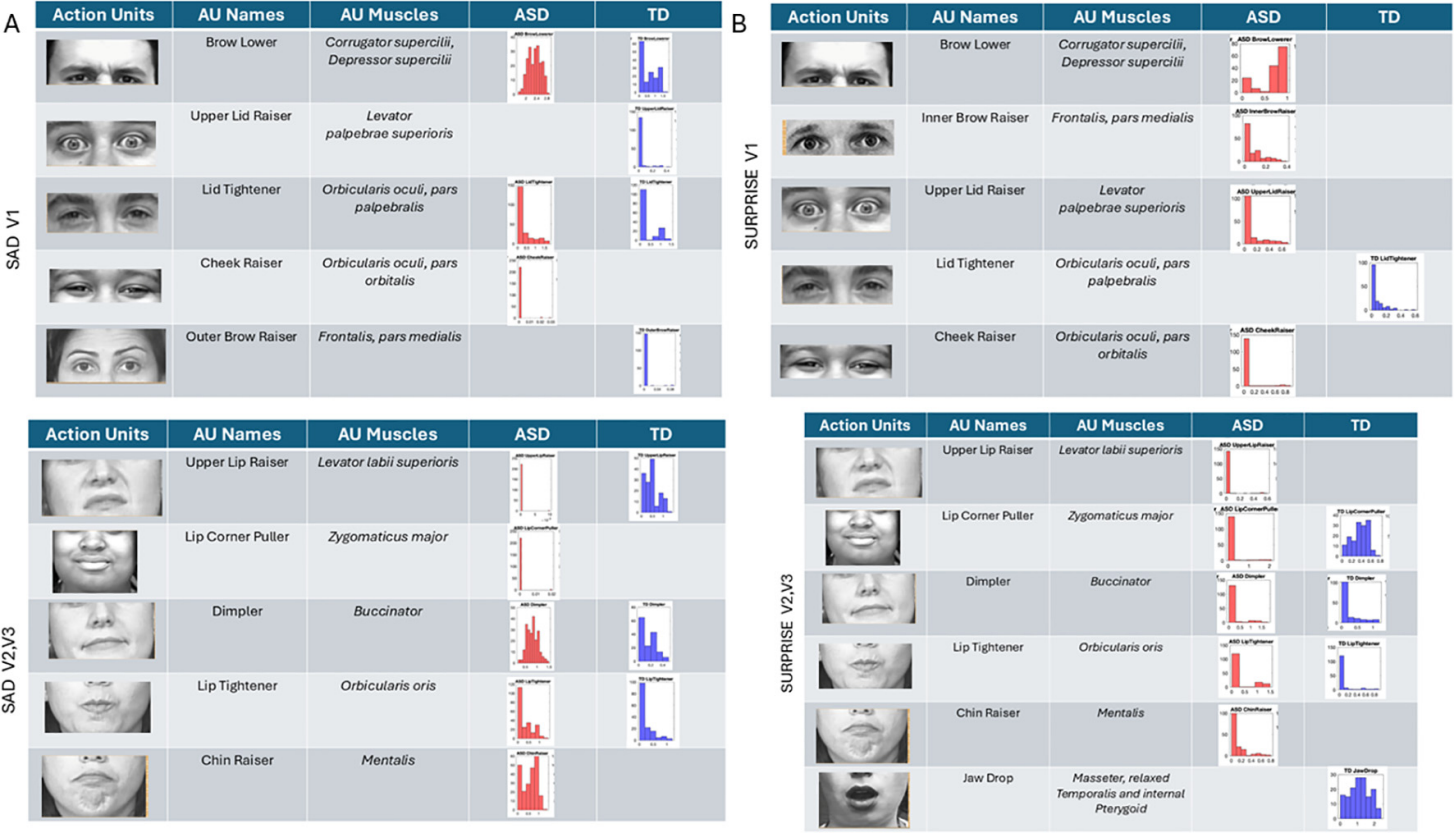
Action units (AUs) for sad **(A)** and surprise **(B)**. As with the other micro-expressions and resting state, several AUs in V1 were commonly present in both groups, yet their intensity differs in ranges and distribution shapes (see text). In the sad micro-expression, the upper lid raised, and outer brow raiser was absent in ASD, but the cheek raiser (key to the happiness micro-expression) was instead present in ASD but absent in TD. For V2 and V3 subregions, in the sad micro-expression, the TD did not have AUs for the lip corner puller and chin raiser, which were both present in ASD. All others were common to both groups but differed in intensity distribution and ranges. The lip tightener common to both groups had comparable intensity. For the surprise micro-expression, more AUs were present in ASD than TD for V1 with complementary patterns. Lid tightener was present in ASD but absent in TD, while brow lower, inner brow raiser, upper lid raiser, and cheek raiser were activated in ASD but not in TD. Subregions V2 and V3 had overlapping AUs for lip corner puller, dimpler, and lip tightener, yet their distributions of intensity differ. ASD had AUs for upper lip raiser and chin raiser present, but these were not present in TD. Lastly, jaw drop, which is common in the surprise micro-expression, was present in TD but absent in ASD, which instead activated chin raiser. ASD, autism spectrum disorder; TD, typically developed.

Sad micro-expressions (**Figure 11A**) revealed in V1 the brow lower AU with much higher intensity values in ASD. Upper lid raiser was absent from ASD, but both groups had lid tightener with comparable distributions of intensity values across participants. In ASD, the cheek raiser was present with low intensity and absent in TD. Subregions V2 and V3 showed the lip corner puller and chin raiser in ASD, but these AUs were absent in TD. The outer brow raiser was absent in ASD but present in TD. In subregions V2 and V3, upper lip raiser was present but much less intense in ASD than in TD. The dimpler was more intense in ASD than in TD, and the distribution of intensities across participants was different, with a much narrower and lower value range in TD.

Surprise (**Figure 10B**) in subregion V1 had brow lower, inner brow raiser, upper lid raiser, and cheek raiser in ASD, but these AUs were absent in TD. TD showed lid tightener, but this AU was absent in ASD. For regions V2 and V3, jaw drop (common in surprise micro-expressions) was present in TD but absent in ASD, along with upper lip raiser. Other AUs were present in both groups but with different levels and distribution ranges of intensities. The lip corner puller was far more distributed in TD than ASD, but ASD had some outliers with high-intensity values, a trend also observed in the lip tightener. The dimpler was present in both and had a comparable distribution of intensity values. Chin raiser was only present in ASD (complementing jaw drop that was only present in TD).

### 3.4 In non-speakers, ASD-HS with apraxia is separable from ASD-HS

**Figure 12:**
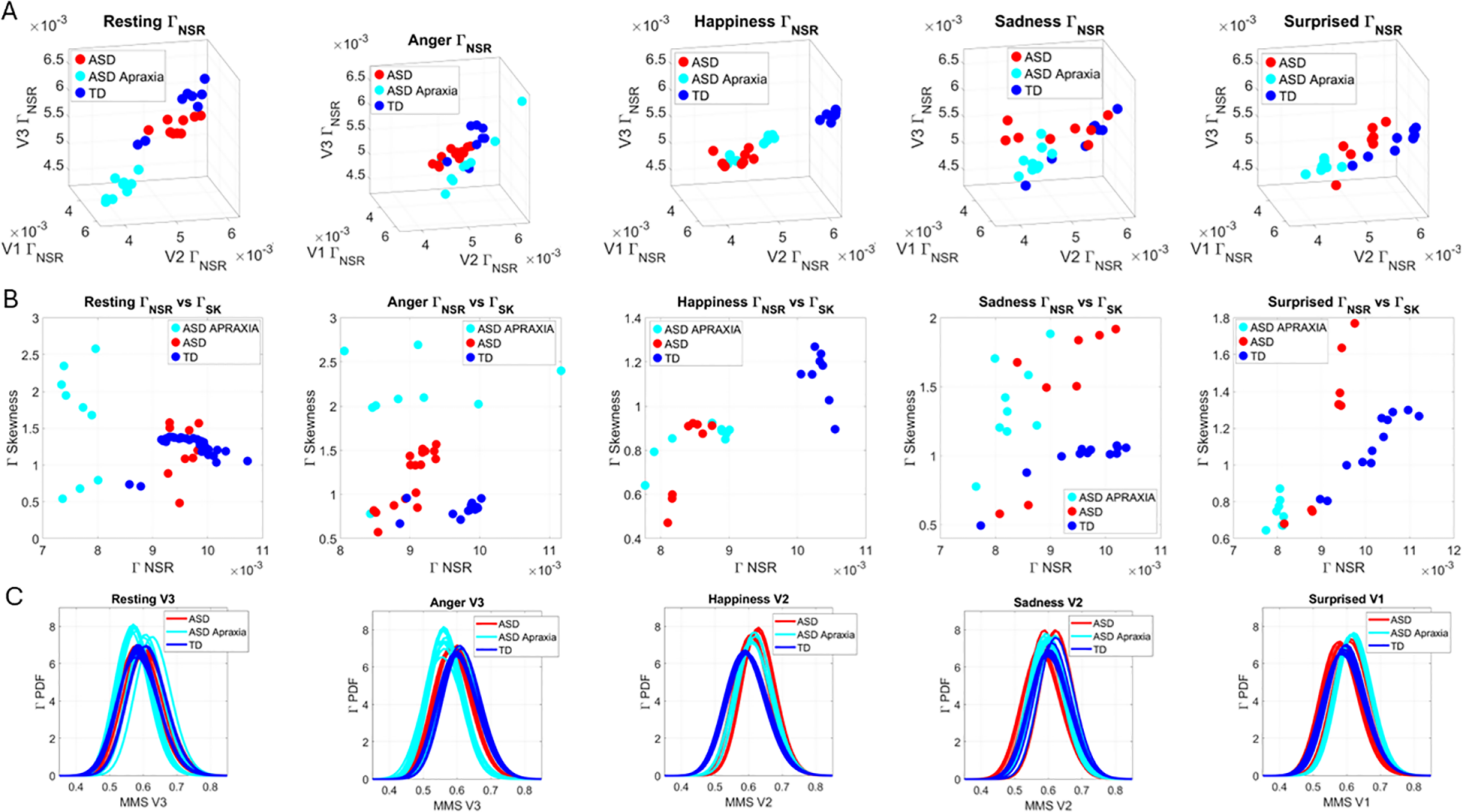
Comparison between non-speaker ASD-HS and those with the additional apraxia diagnosis. **(A)** The Gamma NSR parameter space for resting, anger, happiness, sadness, and surprise tested in this subset of the groups. **(B)** The Gamma NSR scalar quantity *vs.* the Gamma skewness scalar quantity parameter plane with the scatters colored coded according to the three groups. Notice the departure of the apraxia subgroup from the others. **(C)** Selected empirical Gamma PDFs for different subregions of the face. ASD, autism spectrum disorder; HS, high support.

Across resting, anger, happiness, sadness, and surprise, the subset of non-speaker participants with apraxia diagnosis differed significantly from the subset of non-speaker participants with the ASD diagnosis alone. This can be appreciated in **Figure 12A** where we depict the Gamma NSR corresponding to V1, V2, and V3 facial regions and in **Figure 12B** where we show the parameter plane of the Gamma NSR scalar *vs.* the Gamma skewness scalar obtained from the three subregions. **Figure 12C** shows the empirical Gamma PDFs for selected subregions in each of the facial micro-expressions. In each case, the non-speakers with ASD-HS with apraxia separate from the non-speakers with ASD-HS. **Supplementary Figure 5** shows the pairwise statistical comparisons significant at the 0.01 alpha level for the Mann–Whitney test.

### 3.5 Differentiation between ASD-LS and TD across the seven facial universal micro-expressions

**Figure 13:**
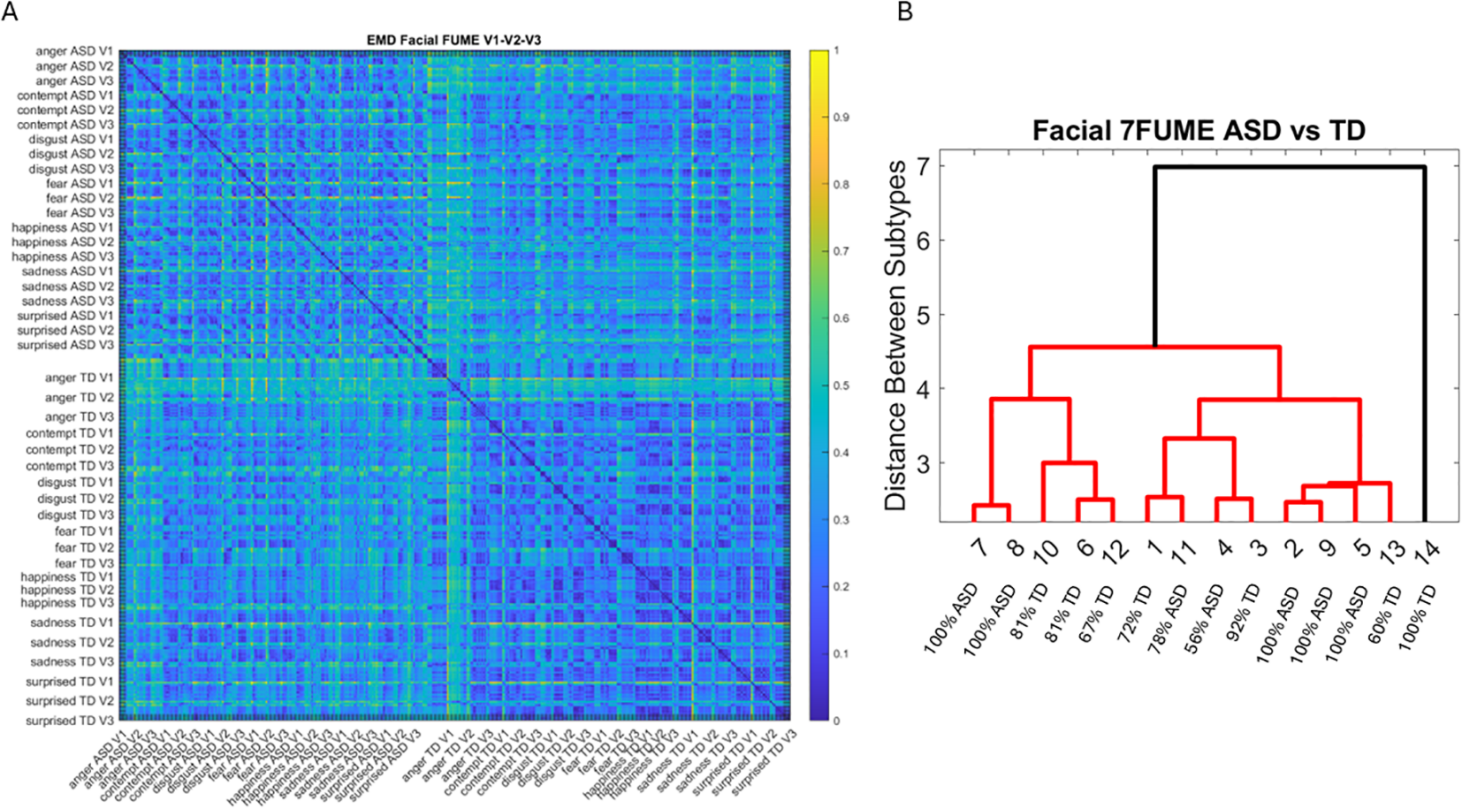
Pairwise EMD for the seven recognized facial universal micro-expressions (anger, contempt, disgust, fear, happiness, sadness, and surprise) **(A)** showing more uniformity (similarity) of distributions in the TD groups and distribution differentiation between ASD-LS group and TD. **(B)** Tree clustering according to 14 subtypes (2 groups × 7 micro-expressions) with the percentage composition of each cluster. EMD, Earth Mover’s Distance; TD, typically developed; ASD, autism spectrum disorder; LS, low support.

The empirical distributions of speed MMSs derived from the positional pixel trajectories in the points of the V1, V2, and V3 regions in ASD-LS (participants requiring a lower level of support and having speaking abilities) were compared to those of TD controls close to their age. The results are shown in **Figure 13A** using the normalized EMD metric values for each pairwise comparison. The structure of this matrix reveals the differences between regions and participating groups. Within each group and facial region, we appreciated the structure of the matrix and saw the self-emerging boundaries of higher values of standardized EMD (denoting higher differences in distribution) with an overall higher similarity within the TD group than within the ASD-LS group (see the more prevalent blue hues denoting similarity in distributions of speed peaks in the standardized MMSs). **Figure 13B** shows the subtypes emerging from the 14 clusters spanned by the two groups and seven facial micro-expressions according to the cluster analysis. The composition of the 14 clusters grouping ASD or TD participants across the leaf of each tree branch was also reported according to the higher percentage of the group for each leaf.

### 3.6 TD composition and non-speaker ASD-HS parental differences

**Figure 14:**
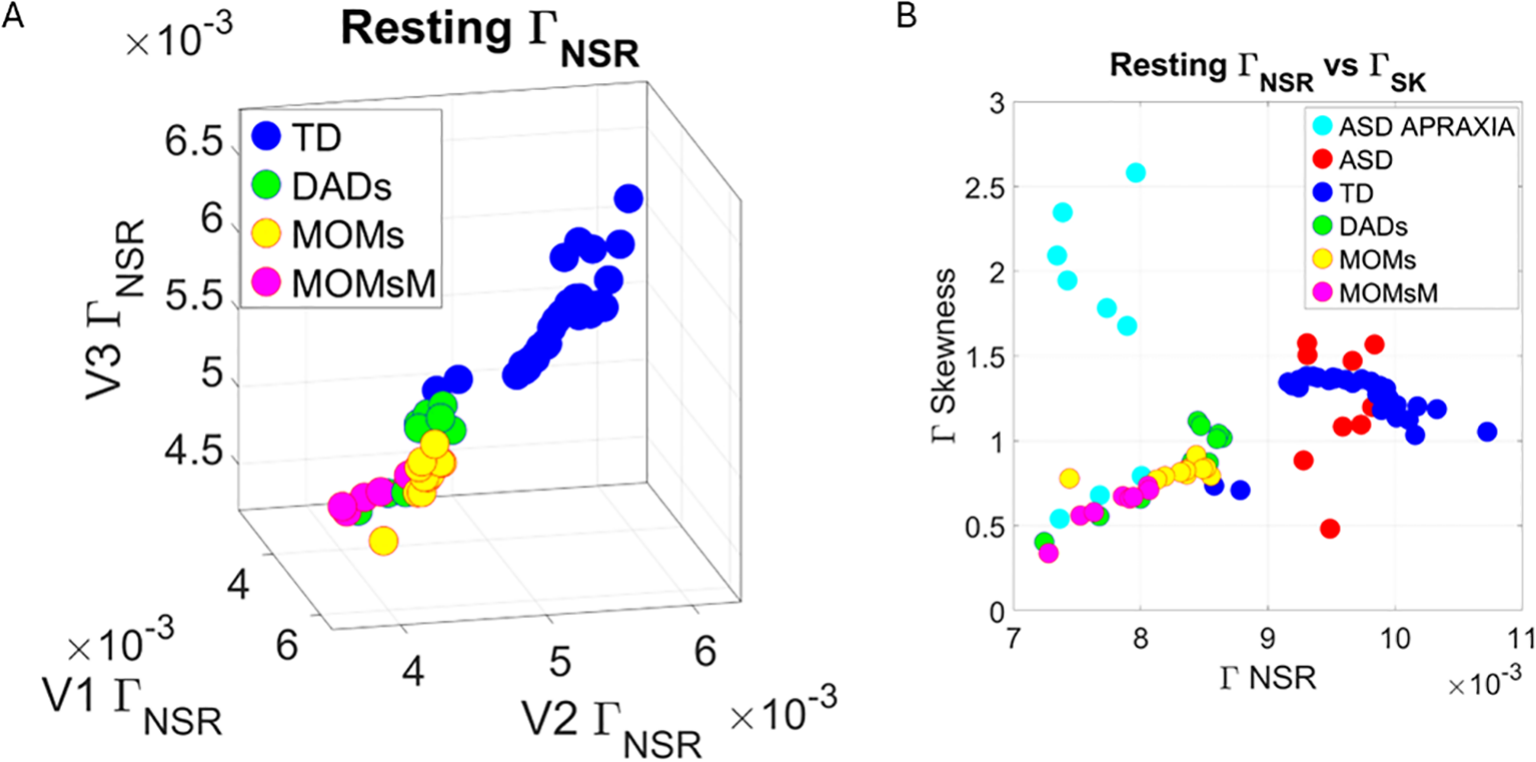
Analysis of TD group labeling the parents of ASD-HS and ASD-HS apraxia **(A)** On the $\Gamma_{NSR}$ space, according to moms, dads, and moms who reported autoimmune disorders and other neuropsychiatric conditions (acquired as adults). Notice the separation and clustering of each subtype departing from other TDs including those of comparable ages. **(B)** Parameter plane of $\Gamma_{NSR}$ scalar quantity *vs.* skewness quantity also shows the ASD-HS and ASD-HS apraxia relative to other TDs. Notice the uniqueness in patterns of some participants with ASD-HD apraxia. TD, typically developed; ASD, autism spectrum disorder; HS, high support.

We compared the Gamma NSR ( $\Gamma_{NSR}$ ) across each of the facial subregions in **Figure 14A** for all TD controls and color-coded the TD parents of non-speaker ASD-HS participants. This comparison revealed that most parents lie within a different subregion of this parameter space, away from most other TD participants of comparable age. Their speed peaks from the standardized MMSs showed lower levels of noise (lower variability), denoting some commonality among this random draw of this population. Furthermore, they self-clustered into dads, moms, and moms with neuropsychiatric conditions (MOMsM). When superimposing the non-speaker ASD-HS and non-speaker ASD-HS with apraxia on the $\Gamma_{NSR}$ *vs*. the Gamma Skewness parameter plane of **Figure 14B**, we appreciated that the NSR levels of the ASD-HS apraxia were comparable to those of their parents. There were higher skewness values for 6/9 participants in this ASD-HS apraxia group, denoting higher speed (faster) deviations from the Gamma mean, a feature that seems to be within a uniquely higher range for this subset of the group.

## 4 Discussion

This work aimed at characterizing the nuances of facial emotional micro-movements using a brief and simple assay under the guidance of instructed emotional facial movements in micro-expressions. Using new personalized methods that do not *a priori* assume theoretical distributions, we wanted to better understand hidden aspects of facial motor control in autistic individuals. These participants spanned from lower to higher levels of support. The study included ASD-HS non-speakers who communicate through various augmented communication methods. To that end, we designed a research data acquisition app that requires very little effort and is brief. The acquisition step offers instructions amenable to being deployed outside the lab during natural activities. These included activities at their school, at a social event where we randomly sampled people in both the TD and ASD groups, and at various clinical settings (studios) where they received therapies.

### 4.1 Highly dysregulated patterns in ASD at rest

The resting state activity had elevated levels of noise across the ASD group, with increasing trends for ASD-HS non-speakers and highest ranges for non-speakers of ASD-HS with a diagnosis of apraxia. The latter is an interesting subset because most if not all ASD-HS participants have a disconnect between the movement plan and the execution of their intended motions. However, in the subgroup with the specific apraxia diagnosis, their baseline dysregulation must be visible to the extent that it reaches the level of visual detection and be diagnosed as an additional disorder. In this sense, resting state noise in facial micro-movements derived from the speed parameter seems to be informative of increasing levels of support across ASD. This result, in the realm of facial micro-movements, is congruent with prior results from our lab involving body micro-movements. Specifically, it is consistent with results concerning excessive motor noise during resting state fMRI reflected in the head motions’ variability (35), including increasing trends with the use of psychotropic medications (36) and general involuntary motions at rest in ASD (29, 38). It is also consistent with increasing random noise in the speed’s micro-movements of voluntary motions increasing with the level of clinical ASD severity in the context of pointing to communicate a decision and/or pointing to a visually prompted target (22, 29). In both bodies of work, we found that the higher the level of support needed in ASD, the higher the Gamma noise level in the micro-movements of the speed kinematic parameter, with increasing trends as the population ages (29, 38, 39).

Within the framework of facial micro-movements, this is the first time that we saw the Gamma noise profiles and can further appreciate the correlation of increasing levels of random noise in the speed motion variability with increasing levels of needed support that have visible apraxia at the highest end. Although evaluating the 3D gaze data from OpenFace outputs will be reserved for a future study, prior work in the field has revealed elevated oculomotor randomness in ASD with increasing trends in noise levels that, as in the pointing case, increase with the level of severity (68). Under these conditions, the type of eye–hand coordination required to deploy the arm linkage (with high degrees of freedom to control) forward to bring the finger to accurately point to a visually prompted target, and then backward to rest, must be very challenging for these individuals. We know, from studying goal-directed movements in the context of deafferentation, that in the absence of reafferent feedback sensation from micro-movements, the neural (surface EEG) correlates of directional motion intent reflect a much higher cognitive load than controls who have proper micro-movement reafferent feedback (69). It is likely that with excessive random noise in the reafferent feedback code, the participants with ASD also experience a higher cognitive load in that they would have to pay attention and be highly aware of activities that typically transpire largely beneath awareness. In this sense, the facial micro-movements activity may reflect the type of dysregulation that a system overburdened with such taxing states is bound to experience.

The present results were derived using the same unifying statistical platform for individualized behavioral analysis that we have used in previous work involving biorhythmic activity data from wearable devices and pose estimation using computer vision techniques (Statistical Platform for Individualized Behavioral Analysis (SPIBA); 70, 71), which have the potential to link, across the population, multiple levels of micro-movements’ noise speed with levels of facial apraxia. They can also reveal issues with eye–hand coordination during pointing behavior to communicate a decision or to point to a visually prompted target. Together, these results have implications for the design and deployment of augmented communication methods. Any communication technique developed for ASD individuals will need to consider these elevated noise levels in the speed parameter to design regulatory support aimed at dampening the motor noise during their clinical therapy and/or school-teaching sessions. Of the activities that we examined under this digital lens, we found that the resting state maximally captured disparities across the cohort. This simple assay may indeed provide the type of information that we need to estimate how regulated a system is, in the precise sense of assessing its level of volitional control. The level of control of the facial and body micro-movements at rest may be informative of individualized levels of overall motor control in flux. It may also help us derive individualized indexes of motor control reflecting the level of agreement between mental intent and physical execution of the intent—an aspect of motor control that is uniquely different in ASD.

The motor noise, which shifts dynamically at the output level as activities of daily life carry on (30, 64), may also serve as a proxy of the quality of feedback that a person’s Central Nervous System (CNS) is receiving from the Peripheral Nervous System (PNS), informing the CNS of ongoing activity, even at the sub-second time scale. As such, the level of regulation of the system and the level of smoothness in action execution (matching the intended plan of the action) may be reflected, at a micro-level, on the facial activity. At one extreme, we have levels of noise and speed MMS distribution skewness that correspond to neurotypical levels. In stark contrast at the other end, we have the largest departure from neurotypical levels on the visibly detectable apraxia. In such cases, the intended plan visibly mismatches the action execution, and even an observer, like a speech therapist giving this diagnosis of apraxia, can detect the mismatch relative to the expected neurotypical levels. Based on these results and the body of knowledge that we have accumulated over a decade of work, we posit that the stochastic signatures of the facial speed micro-movements data may indeed provide a window into the levels of feedback noise, the level of dysregulation, and the associated levels of needed support in ASD.

### 4.2 Presence of action units in ASD but differences in intensity ranges and distributions

Across ASD, we found AUs present underlying the speed micro-movements derived from positional trajectories of the 68 points in the grid that we extracted from videos using OpenFace algorithms. Contrary to the assumption that ASD individuals do not have emotions or lack empathy, we found that they indeed engage (on command) the universal AUs across the face, across subregions V1, V2, and V3 of the digital grid. They, however, do so with different ranges of intensity than those captured in the TD group. As such, the variations in speed amplitudes of the micro-movements from facial universal micro-expressions associated with emotions operate at unexpected stochastic ranges. We posit from these results that folks observing these ranges to screen social engagement and emotions seem to miss these ranges amid rapidly changing social dynamics. It is possible that the expected values of such ranges in neurotypicals do not overlap with those of ASD. Since our visual perception largely depends on our sensitivity levels to visual motion and is biased by that prior experience, we may fail to systematically detect such ASD facial speed ranges. In other words, the ASD facial speed ranges may not intersect with our “detection priors” for the ranges of speed that we typically expect. These results suggest that reliance on observation alone is bound to fail in capturing the emotional capacities of the ASD system and miss an opportunity to engage a person in social exchange.

Across all emotional states probed with the standardized micro-movement data type for true personalized assessment, we saw fundamental differences in the ASD facial micro-expressions but the presence of AUs, nevertheless. This suggests systematic engagement with the person providing the instructions to perform the assay and automatic recruitment of AUs on command. In all those brief 5-second tasks, the ASD participant, across all levels of support and spoken abilities, showed the potential to engage in emotional contexts as the facial system recruited relevant AUs, albeit doing so across different levels of intensity and different distribution ranges of intensity values than TD controls. As with the variations in facial speed micro-movements, under those unexpected ranges, the naked eye of an observer, trying to discern emotional states, will surely miss them. This is so because of inherent statistical learning biases and expected values acquired through interactions with other TD people who are most likely operating within those TD ranges.

### 4.3 Potential for discovery of social–emotional communication in ASD

Given the bidirectional nature of facial micro-expressions, namely, the type of close reafferent loops that engage emotions, it is possible that through training of TD diagnosticians, they can learn to better detect these unexpected ranges of both ASD facial speed micro-movements and ASD AUs’ intensities. It appears that clinicians can detect the two extremes, namely, neurotypical ranges and visible apraxia. They would then engage the ASD individual with greater success than currently done. The results from this investigation are indeed encouraging because they bring a new level of awareness about ASD emotions. Although these levels of intensity and noise are shifted from our perceptual radar, they are present nonetheless in the ASD face. Contrary to the current assumptions and subjective opinions, here, we clearly see that the standardized speed micro-movements present in ASD faces can serve to engage TD controls. As the ASD participants engaged the underlying universal AUs across the different regions of the face grid, we were able to capture these ranges with new analytical means. Moreover, we did so by merely using off-the-shelf instruments that most of us carry around these days.

Our work does not require training large models through machine learning algorithms. Instead, we took direct measurements of speed variability across facial regions evoked by simple, brief assays. Through these unobtrusive, highly scalable means, using commercially available tools on the go, we can further explore other avenues to engage the ASD facial system in unprecedented ways. Bringing these (up to now hidden) ranges of ASD emotions to awareness may make it easier for diagnosticians and therapists engaging with ASD fellows to detect them. Co-adapting our ranges and the ASD ranges is indeed possible now under our new personalized statistical techniques.

This approach to autism is a large departure from trying to impose our neurotypical ways on the ASD person while neglecting the capabilities that their coping systems had already developed by the time that they received the ASD diagnosis. Instead of “normalizing” the ASD individual by imposing our ranges of motion and emotions, here, we propose to expand our perceptual umwelt. By learning to identify the ASD perceptual world and augmenting ours with new expected values, we can also improve our detection systems to operate in a truly diversified humanity. Furthermore, mutual awareness of both our ranges of AUs’ intensity and theirs can bring us closer to building social rapport with the ASD person rather than stigmatizing ASD fellows as social outcasts lacking empathy by assuming that they do not have the desire to engage and communicate with others.

The flip side of augmenting our perceptual umwelt is training ASD individuals to become more aware of their own ranges of micro-motions in the first place. Doing so could help them build self-awareness of their facial micro-expressions and, in this way, own them, learn to control them, and then learn to project desirable configurations at will. Connecting the intent to move with the actual speed of micro-movements could thus become a form of therapeutic intervention mediated by the persons themselves rather than top-down imposed externally by another agent. Owning the action and its consequences can bring the autistic individual to a much-needed level of motor autonomy. Achieving this goal of motor autonomy from the bottom up in autism rather than imposing change from a form of top-down control, e.g., through the currently promoted behavioral conditioning/modification stance, would bring to the autistic person the balance between self-autonomy and self-control (8, 64, 72). In turn, this would enhance their socio-motor agency during social and communication exchanges.

This approach to ASD emotions and social potential is in tune with what autistic individuals themselves want, according to interviews conducted in our lab using the ADOS instrument (40, 73). These observational instruments (the ADOS and the DSM), which deem the ASD person incapable of social communication and boasts a deficit model of social interactions, could in fact benefit from pairing the subjective observational criteria with the type of personalized objective quantification techniques that we show here. Training the diagnosticians in ADOS and DSM settings could significantly improve the diagnostic criteria and help eliminate the stigma that subjective opinion creates across research, clinical settings, and society at large (74–76).

### 4.4 Implications for sensory motor differences related to the brainstem in ASD

The present methods could be applied to screen for signs of brainstem neurodevelopmental differences because the facial regions (ophthalmic, maxillary, and mandibular) are innervated by the trigeminal nerves, which, in turn, connect to the brainstem. In this sense, visual, touch, and auditory stimuli could be used to probe the functioning of such regions in the processing of light, light touch/pressure/movements/pain/temperature, and sound, respectively. Indeed, with respect to sound processing, for instance, retrospective studies have suggested that prolonged auditory brainstem response (ABR) latencies are prevalent in neonates who go on to receive a diagnosis of ASD by 3–4 years of age (58). Furthermore, using clinical observation, the earliest sign predictive of autism is motor delay (77, 78) missed by traditional scales (79) but asserted in 87% of those diagnosed with ASD. These could now be probed non-invasively through the facial involuntary, reflexive, and spontaneous motions.

The facial micro-motions captured here on brief videos could help us track, from a very early age, the development of attention (80, 81), reorienting the head and body for motor control and coordination. Throughout the facial structures and functions involving sensory processing by sensory organs in the eyes, ears, nose, and mouth, we could screen the infant system for unexpected differences indicative of early derailment from the neurotypical developmental path. These sensory organs located on the head sample sensory inputs from different modalities and help form spatio-temporal maps required for the development of proper somatic-sensory-motor integration. These are needed during early dyadic social interactions that develop differently in autism (82). Starting with absent or severely distorted body maps and body schemas (83) and following with delayed milestones in reaching, grasping, postural control, gait, and overall social timing (84–88), ASD, which is an umbrella term for many neurodevelopmental disorders (89–92), could be screened with the help of non-invasive digital means such as those used in this study.

Another area of importance in autism is pain. States of pain could be ascertained through facial micro-movements, a feature that we have recently found present in autistic individuals at rest (52, 53). Sensory hyper- or hypo-sensitivities are common in ASD (90%) and affect all senses (93), inclusive of kinesthetic, pain and temperature reafference, proprioception (6), interoception, and vestibular integration (94, 95). Some are recognized by the diagnostic criteria, and many can be traced to malfunctioning of the brainstem (96), including sensory motor integration, centrally organized by the superior colliculus, the locus coeruleus, the raphe nucleus, and the olivary systems (97–100). Prolonged sensory processing delays are consistent with the findings of extensive literature documenting ABR abnormalities in ASD at various ages. Histological study has found the auditory hindbrain (including the superior olive and inferior colliculus) to have significantly fewer neurons, while surviving neurons have smaller and dysmorphic cell bodies (101, 102). Furthermore, white matter volume in the brainstem of children with ASD is inversely proportional to motor performance (103, 104). Facial features, including the eyes’ gaze direction, head orientation, and mouth-voice analysis, are part of our current studies involving non-speakers requiring high support and deploying the means presented in this paper. These new technologies enable remote use, doing assessments from the comfort of their homes within a participant-centered paradigm. Indeed, the face can be a proxy of states of dysregulation ubiquitous in this population and help us identify ways to regulate the system and relieve it from the cognitive load of having to consciously monitor all aspects of motor control, coordination, and sequencing in activities of daily living that typically occur automatically and largely beneath awareness.

### 4.5 Caveats and future steps

The subset of participating parents was modest. However, their signatures across the facial universal micro-expressions probed in this study revealed unique characteristics. Their features were closer to the stochastic signatures of their offspring than to those of controls around their age. This result is consistent with prior work in motor control biomarkers involving the upper body, during voluntary pointing behavior (28, 29). In those previous studies from our lab, parents of ASD participants showed motor signatures that separated them from age- and sex-matched TD adult controls—as their standardized speed micro-movement signatures were closer to those of their offspring. As with this previous work, here, we point out the possibilities of genetic overlap and/or motor mimicry. The latter could emerge from the efforts by parents to communicate and build rapport with their children over years of interactions.

The study of 126 participants may seem adequate, but given the broad spectrum of variability in ASD—as shown here for the stochastic ranges that we unveiled—it will be important to considerably scale up this research. The present study provides proof of concept that we can do so using simple and brief apps. In future iterations of this study, we plan to gamify the interactions, so we can collect brief data samples in more naturalistic settings, particularly making it more attractive for children of school age.

Our work offers new analytics that do not require machine learning but rather sample directly the empirical distributions corresponding to a person’s individual levels of facial speed noise. Indeed, we see a whole family of PDFs for each participant, starting with the resting state and sweeping through other distributions for anger, disgust, contempt, happiness, sadness, and surprise. With the exponential rise of autism and its absorption of other developmental disorders, we may have to altogether create more emotions to cover those of the autism spectrum. The universality of those micro-expressions, which Paul Ekman helped define, stops with this side of the human spectrum. As such, we do fail to recognize autistic emotions and have profound deficits in socially engaging and communicating with autistic individuals. How can we close this gap?

## 5 Conclusions

Discerning such important research questions in future studies may lead us to learn more about new ways to successfully connect and co-adapt with the autistic motor system—as most parents likely have done. Such an approach to autism would also help broaden our own perceptual ranges and build the proper perceptual umwelt to communicate at a very basic level with the ASD world, particularly the world of non-speakers, which remains such a mystery to science.

The means that we introduced in this study can certainly help us deploy large-scale studies to expand on our results and to pursue new questions involving the fascinating world of ASD social communication and emotional exchange.

Note 1. Vanessa Van Edwards’s facial micro-expression description.
Note 2. GitHub site for OpenFace.
Note 3. Action units used in the study from OpenFace.

## Data Availability

The de-dentified digital data from the raw videos supporting the conclusions of this article will be made available by the authors, without undue reservation.
