# Supplementary material for: Hidden social and emotional competencies in autism spectrum disorders captured through the digital lens
Source: Front Psychiatry. 2025 Apr 7;16:1559202. doi: 10.3389/fpsyt.2025.1559202 (PMC12009872; doi:10.3389/fpsyt.2025.1559202)
Supplement: Supplementary file 1 [file SupplementaryFile1.docx]

Supplementary Material

# Supplementary Figures


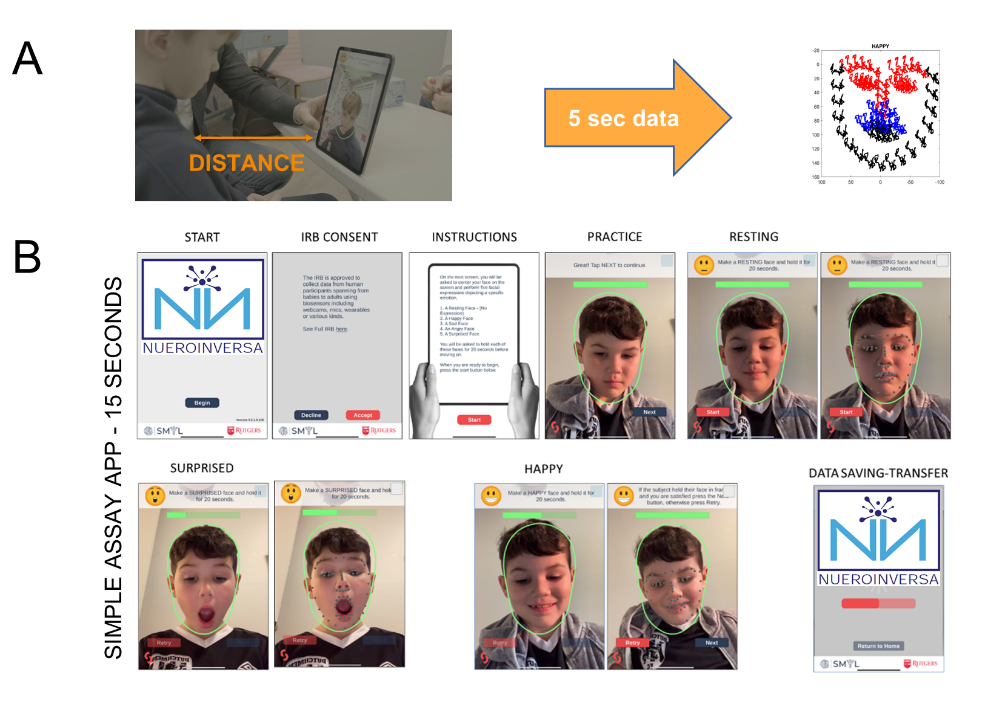


**Supplementary Figure 1.** Data acquisition using an iPad (also possible with an iPhone) (A) The app uses a standardized distance from the person via a green circle which is used to comfortably fit the face inside and collects 5 seconds worth of video. (B). Sample assay used to practice, then resting (5seconds), surprised (open mouth and raise eyes) and happy (smile and raise cheeks)


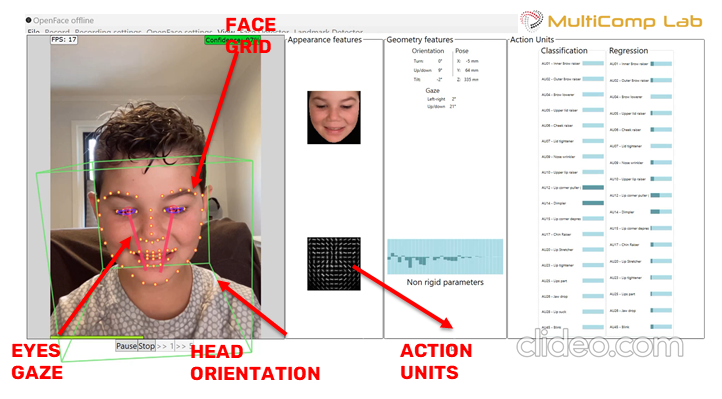


**Supplementary Figure 2.** Output from Open Face software estimating parameters from the 5 second video: face grid with 69 points, 3D eye gaze and head orientation (not used in this paper) and Action Units (used in the analysis)


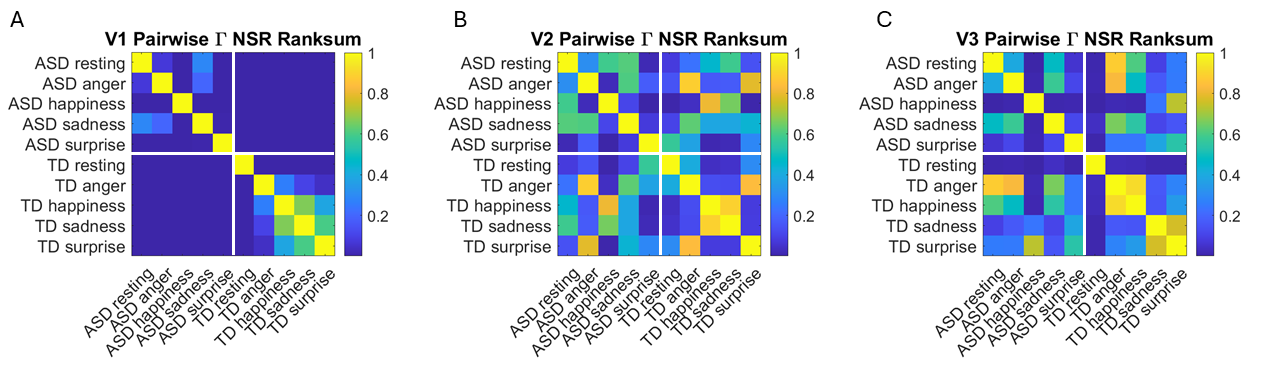


**Supplementary Figure 3.** Results from pairwise Wilcoxon ranksum test statistics (equivalent to the Mann-Whitney U-test) for ASD vs. TD inclusive of micro-expressions during the resting state *vs*. micro-expressions for anger, happiness, sadness and surprise. Area V1 was consistently significantly different between TD and ASD participants across the resting state and all other micro-expressions (taken pairwise, p < 0.01.) Furthermore, in V1, within each of the cohorts, the resting state micro-expressions were significantly different from all other states. In the ASD group, happiness and surprise also differed significantly from all other micro-expressions (p < 0.01). In the TD group, anger vs. surprise was significantly different at p < 0,01 and sadness vs. anger was significantly different at p < 0.05. In areas V2 and V3 statistical differences followed more complex patterns.

In V2, TD at resting state differed from all ASD micro-expressions but only significantly for resting and happiness at p < 0.01. TD anger and surprised differed significantly from ASD happiness at p < 0.01. Within the TD group, resting differed from happiness and sadness, as did happiness and sadness relative to surprise, all at p < 0.05. Within the ASD cohort, anger differed from happiness and happiness from anger at p < 0.01. Surprise in ASD differed from all micro-expressions with significance relative to rest and happiness, p < 0.01.

In V3, the resting micro-expressions in ASD differed from all micro-expressions in TD (p < 0.01). ASD happiness differed significantly from TD anger and TD happiness (p < 0.01). Within the ASD cohort, ASD happiness differed from resting, anger, sadness and surprise (p < 0.01). ASD surprise differed from all micro-expressions with significance at p < 0.01 for happiness and p < 0.05 for resting, while anger and sadness did not reach significance relative to surprise.
